# Toll-like Interleukin 1 Receptor Regulator Is an Important Modulator of Inflammation Responsive Genes

**DOI:** 10.3389/fimmu.2019.00272

**Published:** 2019-02-28

**Authors:** Mohammad Abul Kashem, Hongzhao Li, Nikki Pauline Toledo, Robert Were Omange, Binhua Liang, Lewis Ruxi Liu, Lin Li, Xuefen Yang, Xin-Yong Yuan, Jason Kindrachuk, Francis A. Plummer, Ma Luo

**Affiliations:** ^1^Department of Medical Microbiology and Infectious Diseases, University of Manitoba, Winnipeg, MB, Canada; ^2^JC Wilt Infectious Diseases Research Centre, National Microbiology Laboratory, Winnipeg, MB, Canada; ^3^Department of Biochemistry and Medical Genetics, University of Manitoba, Winnipeg, MB, Canada; ^4^National Microbiology Laboratory, Public Health Agency of Canada, Winnipeg, MB, Canada

**Keywords:** TILRR, NFκB pathway, pro-inflammatory cytokine/chemokine(s), innate immune response, inflammation, microbial infection

## Abstract

TILRR (Toll-like interleukin-1 receptor regulator), a transcript variant of FREM1, is a novel regulatory component, which stimulates innate immune responses through binding to IL-1R1 (Interleukin-1 receptor, type 1) and TLR (Toll-like receptor) complex. However, it is not known whether TILRR expression influences other genes in the NFκB signal transduction and pro-inflammatory responses. Our previous study identified FREM1 as a novel candidate gene in HIV-1 resistance/susceptibility in the Pumwani Sex worker cohort. In this study, we investigated the effect of TILRR overexpression on expression of genes in the NFκB signaling pathway *in vitro*. The effect of TILRR on mRNA expression of 84 genes related to NFκB signal transduction pathway was investigated by qRT-PCR. Overexpression of TILRR on pro-inflammatory cytokine/chemokine(s) secretion in cell culture supernatants was analyzed using Bioplex multiplex bead assay. We found that TILRR overexpression significantly influenced expression of many genes in HeLa and VK2/E6E7 cells. Several cytokine/chemokine(s), including IL-6, IL-8 (CXCL8), IP-10, MCP-1, MIP-1β, and RANTES (CCL5) were significantly increased in the cell culture supernatants following TILRR overexpression. Although how TILRR influences the expression of these genes needs to be further studied, we are the first to show the influence of TILRR on many genes in the NFκB inflammatory pathways. The NFκB inflammatory response pathways are extremely important in microbial infection and pathogenesis, including HIV-1 transmission. Further study of the role of TILRR may identify the novel intervention targets and strategies against HIV infection.

## Introduction

**Figure 1:**
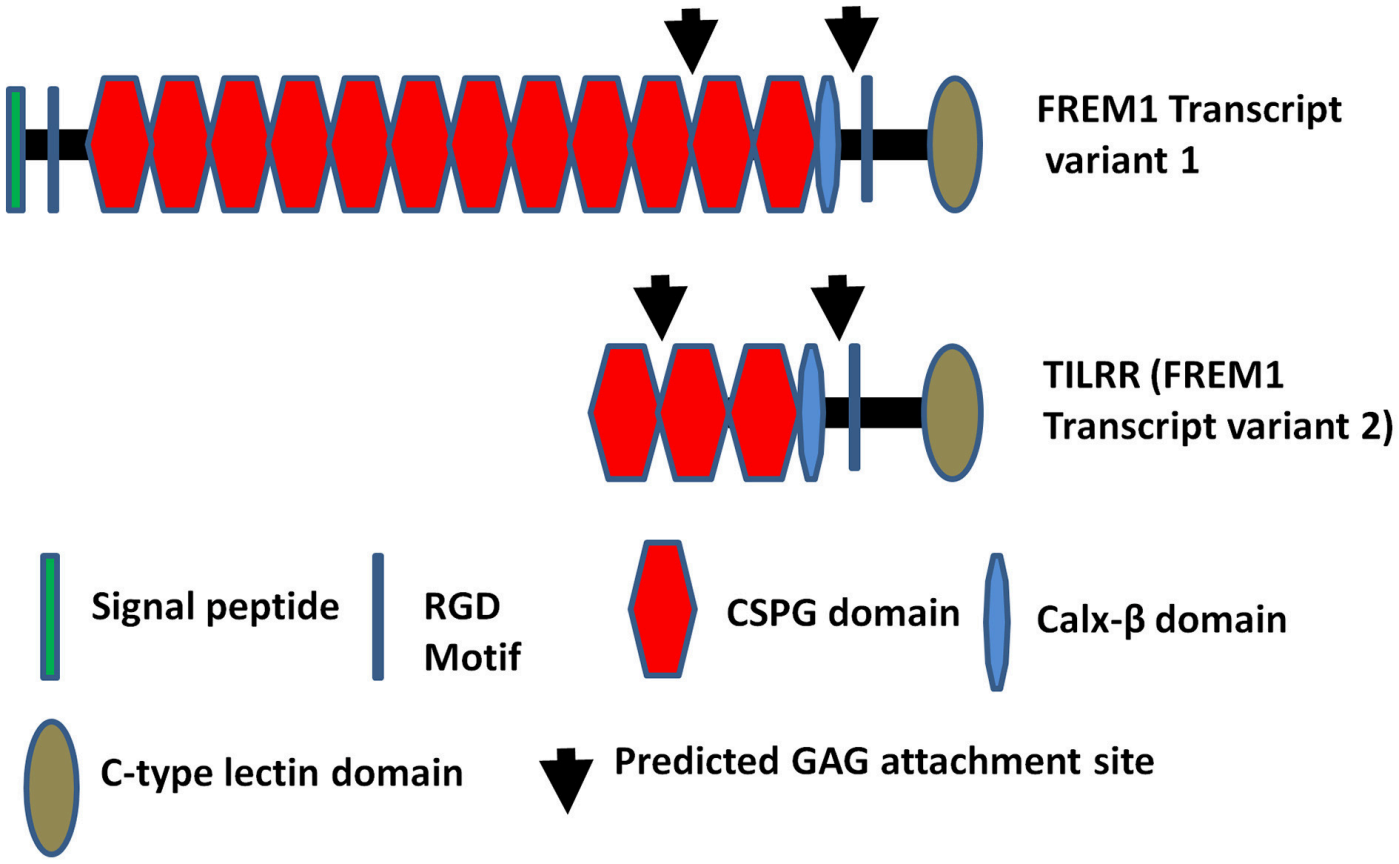
Diagram of FREM1 variants. Full length FREM1 transcript variant 1 (top) and FREM1 truncated variant 2 (also called TILRR) (bottom); CSPG, chondroitin sulfate proteoglycan; RGD, arginine-glycine-aspartic acid; GAG, glycosaminoglycan.

FREM1, a Fras Related Extracellular Matrix 1 protein, originates from epithelial and mesenchymal cells (1, 2). It is widely expressed in the developing embryo in the region of epithelial/mesenchymal interaction (3) and the basement membrane zone of hair follicles (4). It is also highly expressed in the cervix, kidney and small intestine compared to other tissues in human (5). A significant number of studies have shown that mutations in the FREM1 and its splice variants are associated with MOTA (Manitoba-oculo-tricho-anal) syndrome (6–11), BNAR (Bifid nose, anorectal and renal agenesis) syndrome (10, 12), prenatal hydrocephalus and shortened limbs (13), metopic craniosynostosis (14), and congenital diaphragmatic hernia (15, 16) in human and mice. Recent study demonstrated that FREM1 is also associated with facial morphology in human (17). Previously, we identified FREM1 as a novel candidate gene involved in HIV-1 resistance/susceptibility in the Pumwani Sex worker cohort (5). Of over 15 FREM1 splice variants, one has been identified as a toll-like interleukin-1 receptor regulator (TILRR). TILRR was described as a novel regulatory component, which functions as an IL-1R1 co-receptor (18, 19). Recently, it has been shown that TILRR is responsible for the development of cardiovascular disease via aberrant activation of inflammatory genes (20). Structurally, TILRR contains three CSPG (chondroitin sulfate proteoglycan) domains and a RGD (arginine-glycine-aspartic acid) domain that interact with collagen, integrin, and fibronectin (4). It also has Calx-β, C-type lectin (LecC) domains, and two GAG (glycosaminoglycan) attachment sites that bind to IL-1R1 (Figure 1) (5). It has been demonstrated that TILRR amplifies innate immune and inflammatory responses after binding to IL-1R1 and TLR complexes by enhancing the recruitment of MYD88 (Myloid differentiation primary response 88) in the Ras-dependent NFκB signal transduction pathway (19, 21). However, it is not known whether TILRR interacts with or regulates other genes in the NFκB signal transduction and pro-inflammatory responses. The NFκB and pro-inflammatory responses are very important in initiating innate immune responses to pathogens, including HIV infection, and in linking innate and adaptive immune responses. Therefore, it is important to understand the effect of TILRR on genes in the NFκB signal transduction pathway. We hypothesized that TILRR regulates genes in the NFκB signal transduction pathway, directly involved in immune activation and inflammatory responses.

In this study, we used two epithelial cell lines that do not express TILRR mRNA to investigate the effect of TILRR overexpression on mRNA of genes in the NFκB signaling pathway and its effect on several soluble immune mediators. We evaluated the mRNA expression of 84 genes related to the NFκB signaling using PCR arrays and the secretion of 13 soluble immune mediators using a multiplex bead array system.

## Materials and Methods

### Cell Lines and Culture Condition

Our previous study showed that FREM1 mRNA is highly expressed in human cervical cells (5). To study the effect of FREM1 variant TILRR expression on cervical cells we used two model cervical epithelium cell lines, HeLa and VK2/E6E7. RNA-seq analysis and qRT-PCR analysis showed that these two cell lines do not express TILRR mRNA under the cell culture conditions we used in the study. Thus, we can use them to overexpression TILRR to study the effect on other inflammatory response related genes.

HeLa cells (National Institute of Health AIDS reagent program, USA; Catalog# 153) were maintained in Dulbecco's Modified Eagle's Medium (DMEM) (Sigma-Aldrich, D5796) supplemented with 10% FBS (fetal bovine serum; Gibco, Catalog# 12483-020) and 1% Antibiotic-Antimycotic (Gibco, Catalog# 15240062). VK2/E6E7 cells (ATCC, Catalog# CRL2616) were maintained in Keratinocyte-SFM (1X) growth medium (Gibco, Catalog# 10724-011), supplemented with human recombinant EGF (Epidermal Growth factor, 0.1 ng/ml), and BPE (Bovine Pituitary Extracts, 50 μg/ml) (Gibco, Catalog# 37000-015), 0.4 mM CaCl_2_ (Sigma Aldrich, Catalog# C5670) and 1% Penicillin-Streptomycin solution (Gibco, Catalog# 15140-122). Adherent cells in culture were detached from the T175 flask with 0.25% Trypsin-EDTA (Gibco, Catalog# 25200056), and the enzyme was deactivated with sufficient volume of complete DMEM growth medium for HeLa, and DMEM Nutrient mixture F-12 Ham (Sigma Aldrich, Catalog# D8437) supplemented with 10% FBS (Gibco, Catalog# 12483-020) plus 1% Penicillin-Streptomycin solution (Gibco, Catalog# 15140-122) for VK2/E6E7 based on the recommended protocols (ATCC). All the cells were then incubated at 37°C with 5% CO_2_ for 2–3 days until 90–100% confluent.

### Plasmid Constructs, Reagents and Transfection

Both the TILRR-plasmid (GeneCopoeia, Catalog# EX-I2135-68) and Empty vector-plasmid control (GeneCopoeia, catalog# EX-NEG-68) containing a CMV promoter, an ampicillin marker, and a puromycin marker, were used for transfecting cells (Figure S1). PmaxGFP (Lonza, Walkersville, MD, USA) was used as a standard enhanced GFP (Green fluorescence protein) control vector to monitor the transfection efficiency by Flow Cytometry and Confocal Microscopy. EndofectinMax (GeneCopoeia, Catalog# EFM1004-01) transfection reagent was used for the lipid based transfection of cells. Approximately 2.5 × 10^5^ cells/ml were plated into each well of a 12-well culture plate in complete DMEM growth medium (HeLa) or complete Keratinocyte-SFM (1X) (VK2/E6E7) a day before transfection, and incubated at 37°C with 5% CO_2_ for 24 h. Once the cells reached 80–90% confluency, the medium was replaced with antibiotic free fresh growth medium. Co-transfection was performed using different concentration of either TILRR-plasmid (vector+TILRR) (0.25, 0.5, 1.0, or 2.0 μg per well) or empty vector-plasmid (empty vector control) (0.25, 0.5, 1.0, or 2.0 μg per well) in combination with PmaxGFP-plasmid DNA (0.05, 0.1, 0.2, or 0.4 μg per well, respectively; 1:5 ratio) with 2 μl of EndofectinMax transfection reagent following to the protocol recommended by the manufacturer. After incubation at 37°C with 5% CO_2_ for 24 h the effect of TILRR overexpression on mRNA expression of 4 genes was analyzed. The optimized concentration of TILRR-plasmid (1.0 μg/well) or empty vector (1.0 μg/well) in combination with PmaxGFP vector (0.2 μg/well; 1:5 ratio) was used to transfect HeLa and VK2/E6E7 cells with 2 μl of EndofectinMax transfection reagent to analyze the effect of overexpression of TILRR on mRNA expression of NFκB signal transduction pathway related genes and pro-inflammatory cytokine/chemokine(s).

### Confocal Microscopy and Flow Cytometry

Transfected cells expressing eGFP and TILRR protein were visualized using a confocal microscopy imaging system (Zeiss LSM 700, Laser Scanning Microscope). For visualization of overexpressed TILRR protein in transfected cells, the TILRR-plasmid (vector+TILRR) transfected cells (after puromycin selection) and parental control (non-transfected) cells were plated in Glass bottom 6-well plate (MatTek Corporation, Cat# P06G-1.5-20-F) for 24 h. The cells were washed once with 1x PBS, then fixed with 4% paraformaldehyde (pH 7.4) (Electron Microscopy Sciences, Catalog# 15713-S) for 10 min at 37°C. The fixed cells were washed 3 times with 1x PBS, and permeabilized with 0.1% TritonX-100 (Sigma Aldrich, Catalog# X100–100 ml) for 10 min at room temperature (RT). After further washes (3 times with 1x PBS), the cells were blocked with 3% BSA (Sigma Aldrich, Catalog# A9418-10G) containing 0.1% TritonX-100 in 1x PBS at RT for 1 h. Following blocking, the cells were incubated at RT for 3 h with a mixture of two in-house developed mouse monoclonal antibodies (mabs) targeting epitopes of TILRR (F218G1 and F218G5) (2 μg/ml, diluted in 1x PBS containing 0.1% BSA and 0.1% TritonX-100). An isotype control experiment with a mab (2 μg/ml) (F400G3S) specific for a *Chlamydia trachomatis* antigen was conducted in parallel. After washing (three times with PBS-T blocking buffer containing 0.05% TritonX-100 + 1.5% BSA), the cells were stained with Alexa Fluor 647-labeled goat anti-mouse IgG secondary antibody (Invitrogen, Catalog# A-21235) (2 μg/ml, diluted in 1x PBS containing 0.1% BSA and 0.1% TritonX-100) for 45 min at room temperature. As a negative control, we also incubated cells with only Alexa Fluor 647-labeled goat anti-mouse IgG secondary antibody (2 μg/ml) without primary mabs cocktail. Following washes (3 times with PBS-T), the cells were incubated for 5 min with counter stain for nucleus, DAPI (300 nM) (Invitrogen, Catalog# D1306). After staining, the cells were kept in 1 ml of 1x PBS and sealed with Parafilm M (Sigma Aldrich, Catalog# P7793-1EA) and examined with Confocal microscopy imaging system using three color channels for DAPI, FITC and Alexa Fluor 647.

We also quantified overexpression of TILRR protein in transfected HeLa and parental cells by FACS analysis (BD Accuri C6, BD Biosciences). We stained the cells according to the BD Biosciences (California, USA) protocol. Briefly, 5 × 10^5^ HeLa cells from each of the experimental conditions were prepared and washed with 1x PBS containing 2% FCS (fetal calf serum), then incubated with 50 μl Alexa Fluor 647 labeled in-house developed mabs (F218G1 and F218G5) (2 μg/ml, diluted in 1x PBS containing 3% BSA) for 30 min at 4°C in dark (APEX Antibody Labeling kit, Invitrogen, Catalog# A10475). After washing (PBS containing 2% FCS), 100 μl BD permeabilizing solution (BD Biosciences, catalog# 554714) was added. After 10 min permeabilization, the cells were washed twice with 1x Perm/Wash buffer (BD Biosciences, catalog# 554714), and then 50 μl of Alexa Fluor 647 labeled mabs cocktail (F218G1 and F218G5) (2 μg/ml, diluted in 1x Perm/Wash buffer) was further added and incubated for 30 min at 4°C in dark. Finally, the cells were resuspended in PBS containing 2% FCS after two times washes with 1x Perm/Wash buffer and analyzed with BD Accuri C6. In parallel, the cells were also stained with isotype control mab (F400G3S) (2 μg/ml) labeled with Alex Fluor 647. FlowJo Software (Treestar, USA) was used for analysis. Cell viability by FACS was measured using Live/Dead Fixable Red Dead Cell stain (Life Technologies, Catalog# L34971) following the company's recommended protocol.

### Collection of Conditioned Media for Cytokine/Chemokine(s) Assay

The HeLa and VK2/E6E7 cells were transfected with TILRR-plasmid or empty vector-plasmid control as described in the method above. Twenty-four hours after transfection the cells were treated with puromycin dihydrochloride (Gibco, Catalog# A11138-03) for 24 h to remove untransfected cells. The cells were then incubated in serum free DMEM (HeLa) or Keratinocyte SFM (1X) (VK2/E6E7). In parallel experiments, the cells were also incubated with human interleukin-1β (IL-1β; 1 nM) (Sigma-Aldrich, Catalog# I9401) in serum free HeLa and VK2/E6E7 cells medium. The cell culture medium was collected at 1, 3, 6, 15, and 24 h for cytokine/chemokine(s) analysis.

### RNA Extraction, Purification, Quantification, Quality Analysis and cDNA Synthesis

RNA was extracted from cells under different experimental conditions using RLT buffer from RNeasy Mini Kit (Qiagen, Catalog# 74104). Extracted and purified RNA from 5 × 10^5^ cells/experimental condition using RNAeasy Mini Kit according to the manufacturer's instructions. The purified RNA was quantified using a NanoDrop 1000 Spectrophotometer (Thermofisher Scientific, USA), and the *A260:A230* ratio of the isolated RNA was >1.7 and their *A260:A280 ratio* was between 1.8 and 2.0. RNA quality was also assessed with 2100 Agilent^(R)^ Bio-analyzer (Agilent Technologies, USA) using an RNA 6000 Nano LabChip^(R)^ kit (Agilent Technologies, Catalog# 5067-1511), and verified the quality with sharp bands/peaks for both the 18S and 28S ribosomal RNAs. The RIN was ≥7.0 for each sample. The cDNA was synthesized using RT^2^ first strand kit (Qiagen, Catalog# 330404) with 500 ng purified RNA per reaction as recommended by the manufacturer's protocol.

### RT^2^ qPCR Primer Assay and RT^2^ Profiler PCR Array

Real time quantification of TILRR overexpression was done using a commercial RT^2^ qPCR primer assay (Qiagen, Catalog# PPH11469A-200). NFκB signaling pathway expression was quantified using RT^2^ profiler qPCR array (Qiagen, Catalog# PAHS-025Z) and RT^2^ SYBR^(R)^ Green ROX qPCR Mastermix (Qiagen, Catalog# 330523). We also performed RT^2^ qPCR primer assay for 4 selected immune responsive genes, CCL5 (Catalog# PPH00703B-200), CXCL8 (Catalog# PPH00568A-200), IL-6 (Catalog# PPH00560C-200) and TNFα (Catalog# PPH00341F-200), to measure the mRNA transcript expression with similar Mastermix as mentioned above. All primers were purchased from Qiagen. We used 1 μl of cDNA in 25 μl reaction volume. Amplification of cDNA performed in 40 cycles, consisting of initial 1 cycle at 95°C for 10 min followed by 40 cycles, each cycle run at 95°C for 15 s followed by 60°C for 1 min. After 40 cycles, we also performed dissociation curve for all 84 genes and threshold was manually corrected at 0.4. Data were exported and finally organized in Microsoft Office Excel sheet and analyzed by GeneGlobe Data Analysis Centre (Qiagen). Applied BioSystem 7900 HT Fast Real time PCR 96-well standard block (ThemoFisher Scientific, USA) was used for all qRT-PCR analysis.

### Western Blot

A previously published method was used with slight modifications (22). Briefly, SDS-PAGE was conducted using NuPAGE Bis-Tris mini gel electrophoresis protocol. Approximately 1 × 10^6^ cells were lysed with 50 μl RIPA lysis and extraction buffer (Thermo Fisher Scientific, Catalog# 89900), then passed through a QIAshredder column (Qiagen, Catalog# 79654) by centrifugation at 15,000 g for 2 min. The lysate was then prepared and loaded into a NuPAGE 4–12% Bis-Tris 1.0 mm × 10well gel (Thermo Fisher Scientific, Catalog# NP0321BOX). Several monoclonal antibodies were used to detect the TILRR protein including F218, F208, F217, F244, F220, and F237 previously developed in our lab (23). The monoclonal antibodies were diluted in antibody buffer (wash buffer containing 0.5% skimmed milk) to give a concentration of 1 μg/ml for each antibody, and then incubated overnight with membranes at 4°C with shaking. Then, the secondary antibody, goat anti mouse IgG-HRP (Santa Cruz Biotechnology, Catalog# sc-2005) was diluted at 1:5,000 in antibody buffer and incubated with membrane for 1 h at room temperature with shaking. Chemiluminescent detection was performed on a ChemiDoc XRS instrument using Quantity One 4.6.9 software (Bio-Rad). The level of TILRR protein expression was defined as ratio of the band intensity of TILRR to that of GAPDH, and finally normalized to parental cells.

### Bioplex Cytokine/Chemokine(s) Multiplexed Bead Assay

A custom 13-plex cytokine/chemokine panel was used to measure the level of Granulocyte Macrophage Colony Stimulating Factor (GM-CSF), IFNγ, IL-1β, IL-6, IL-8 (CXCL8), IL-10, IL-17A, IFN-γ inducible protein 10 (IP-10), Macrophage Chemo-attractant Protein 1 (MCP-1), Monocyte Inflammatory Protein 1 alpha (MIP-1α), Monocyte Inflammatory Protein 1 beta (MIP-1β), RANTES (CCL5), and TNFα (Table S5 for detailed information). The primary antibodies (mouse) for each cytokine/chemokine were coupled to 1.25 × 10^6^ Bio-Plex Pro™ Magnetic COOH Beads (BioRad, Catalog# MC10053-01) using BioPlex^(R)^ Amine coupling kit (BioRad, Catalog# 171-406001) according to the supplier's instructions. The assay was performed based on Bio-Plex Pro™ assays protocol (BioRad). Briefly, the antibody coupled beads were vortexed and combined at 1:600 dilutions in assay buffer (Bio-Plex Pro™ reagent kit, BioRad, Catalog# 171-304070M). 50 μl of combined beads from all the 13 individual cytokine/chemokine was added in the Bio-Plex Pro™ Flat bottom plate (BioRad, Catalog# 171025001) and washed twice with 100 μl Bio-Plex wash buffer (BioRad, Catalog# 171-304070M) at room temperature (RT). Fifty microliter conditioned media was added to the plate and shaken for 30 s at 1,000 rpm and then incubated for 30 min on plate shaker (850 ± 50 rpm) at RT. The plate was washed twice and 25 μl of detection antibody (1 μg/ml) was added per well, incubated again for 30 min on a plate shaker. Fifty microliter streptavidin-PE conjugate (1x) (BioRad, Catalog# 171304501) was added per well after three washes, and incubated for 10 min at RT. Finally, the plate was washed three times, added 150 μl of assay buffer in to each well, shaken for 10 s and then run by Bio-Plex™ 200 System (Luminex xMAP technology) (Bio-Rad, Canada). Complete growth media (HeLa and VK2/E6E7 cell specific) were used as diluents for Bio-Plex Pro Human Cytokine Standards Group I 27-plex (BioRad, Catalog# 171D50001) and as a background (blank) control. To generate standard curve, we added 50 μl of 4-fold standard dilutions in 6-wells in duplicates and the correlation coefficient (*R*^2^) was being calculated in each experiment to see the linearity of the standard curve. Data were generated by Bio-Plex Manager 6.1 software.

### Venn Diagram, Heat Map Generation

Venn diagram was made by using pyvenn (https://github.com/tctianchi/pyvenn). Heat Map was created using RStudio (https://www.rstudio.com/).

### Statistical Analysis

RT^2^ Primer qPCR assay and RT^2^ profiler PCR array data were analyzed using GeneGlobe Data Analysis Centre (Qiagen) (https://www.qiagen.com/us/shop/genes-and-pathways/data-analysis-center-overview-page/). The mRNA transcript fold changes in threshold cycle (CT) were calculated relative to the CT level of empty vector control. Fold change 1.0 assigned as a baseline control and threshold cycle (CT) assays were performed in three independent experimental replicates. All mRNA transcript data were normalized against HPRT1 housekeeping gene. Graphical presentation of fold change was organized by GraphPad Prism software, version 7.03 (GraphPad Software, Inc. USA). The cytokine/chemokine(s) data were also analyzed by GraphPad Prism version 7.03 and presented as relative to the concentration of empty vector control, and show mean ± SEM of three independent experiments. The final statistical comparisons conducted using student *t*-test with 95% CI, all *p* < 0.05 were reported and indicated using an asterisks' ^*^p < 0.05, ^**^p < 0.01, ^***^p < 0.001, and ^****^p < 0.0001.

## Results

### TILRR Overexpression in Transfected Cells

**Figure 2:**
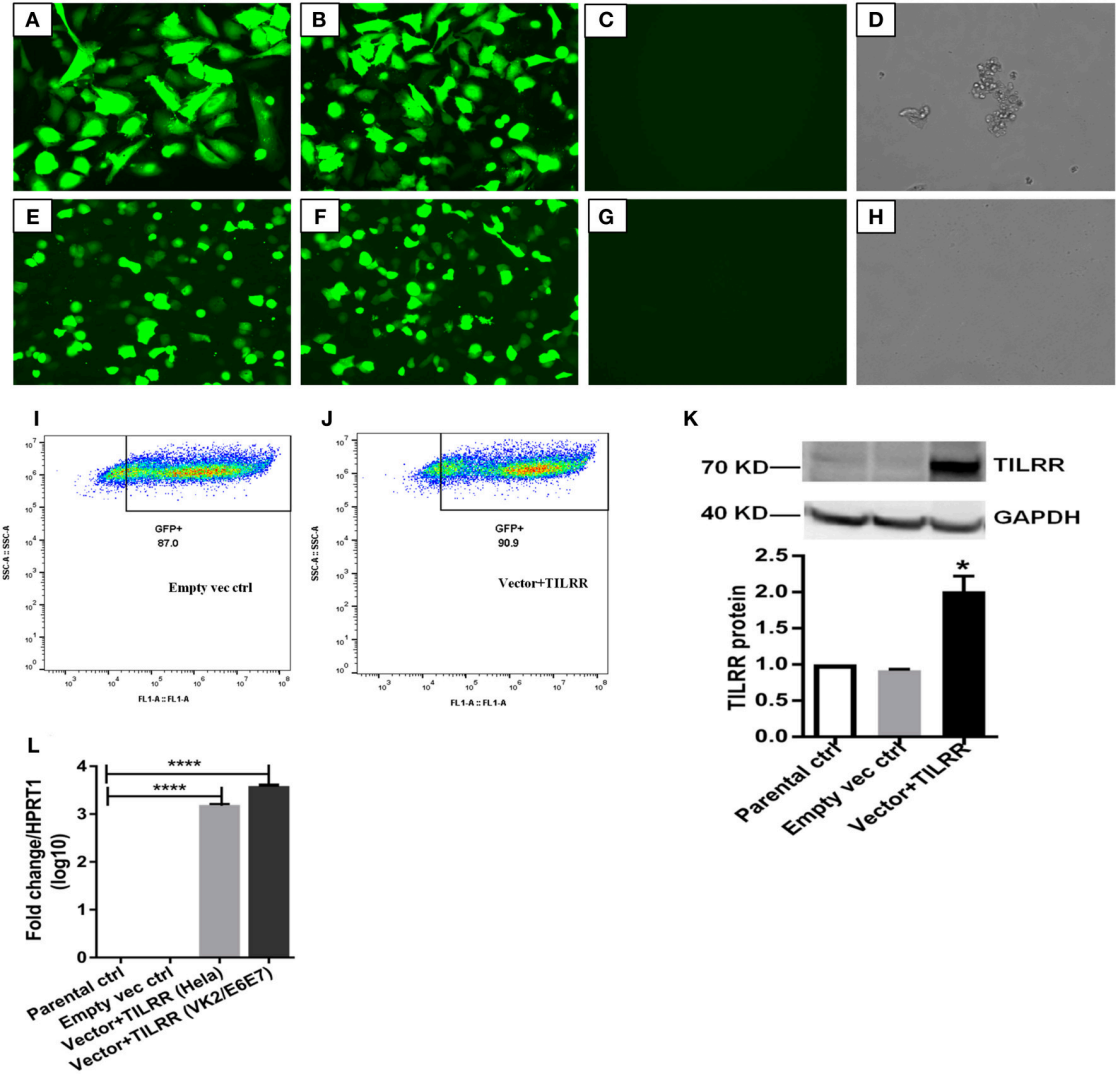
Expression of TILRR in cell lines. **(A–H)** Confocal microscopy of transfected HeLa **(A–D)** and VK2/E6E7 **(E–H)** cells showing eGFP expression following co-transfection with pEZ-TILRR-M68 (vector+TILRR) (1.0 μg/5 × 10^5^ cells) **(A,E)** or pEZ-NEG-M68 (empty vector control) (1.0 μg/5 × 10^5^ cells) **(B,F)** along with PmaxGFP (0.2 μg/5 × 10^5^ cells) and non-transfected control **(C–D,G–H)**. **(I–J)** Flow cytometry quantitation of transfected HeLa cells (I, empty vector control–transfected cells; J, TILRR-overexpressed cells). **(K)** Western blot of TILRR proteins following 24 h post-transfection, as compared to GAPDH control (full length original blots presented in Figure S2). **(L)** Confirmation of the TILRR mRNA transcripts overexpression in both cells using RT^2^ qRT-PCR primer assay and data presented as log10 fold change, which was normalized against HPRT1 housekeeping gene. Student *t*-test with 95% CI performed for the statistical analysis using GraphPad prism version 7.03, all *p* < 0.05 were reported and indicated using an asterisks' ^*^p < 0.05, and ^****^p < 0.0001.

To assess the effect of TILRR overexpression on genes in the NFκB inflammatory pathway, we overexpressed TILRR in HeLa (a cervical epithelial cell-line) and VK2/E6E7 (a normal human vaginal mucosal epithelial cell-line) cells. We transiently transfected HeLa (Figures 2A,B) and VK2/E6E7 (Figures 2E,F) cells with a TILRR expression plasmid that includes a CMV promoter and Puromycin selection marker. Confocal microscopy image analysis showed that cells containing plasmids with either TILRR plus puromycin selection marker (vector+TILRR) or only puromycin marker (empty vector control) were alive after 24 h puromycin dihydrochloride selection as shown by the cells attached to the culture plate with active pseudopods and intact morphology (Figures 2A,B,E,F). Whereas, within the same period of time under puromycin selection the non-transfected cells or the cells that only contain PmaxGFP vector were died, showing complete loss of pseudopodia and detachment from the culture plate with distracted morphology (Figures 2C,D,G,H). Flow cytometry quantification of GFP expressing cells showed that the transfection efficiency was between 87.0 and 90.9% of empty vector control- and TILRR (vector+TILRR)-transfected HeLa cells, respectively (Figures 2I,J).

**Figure 3:**
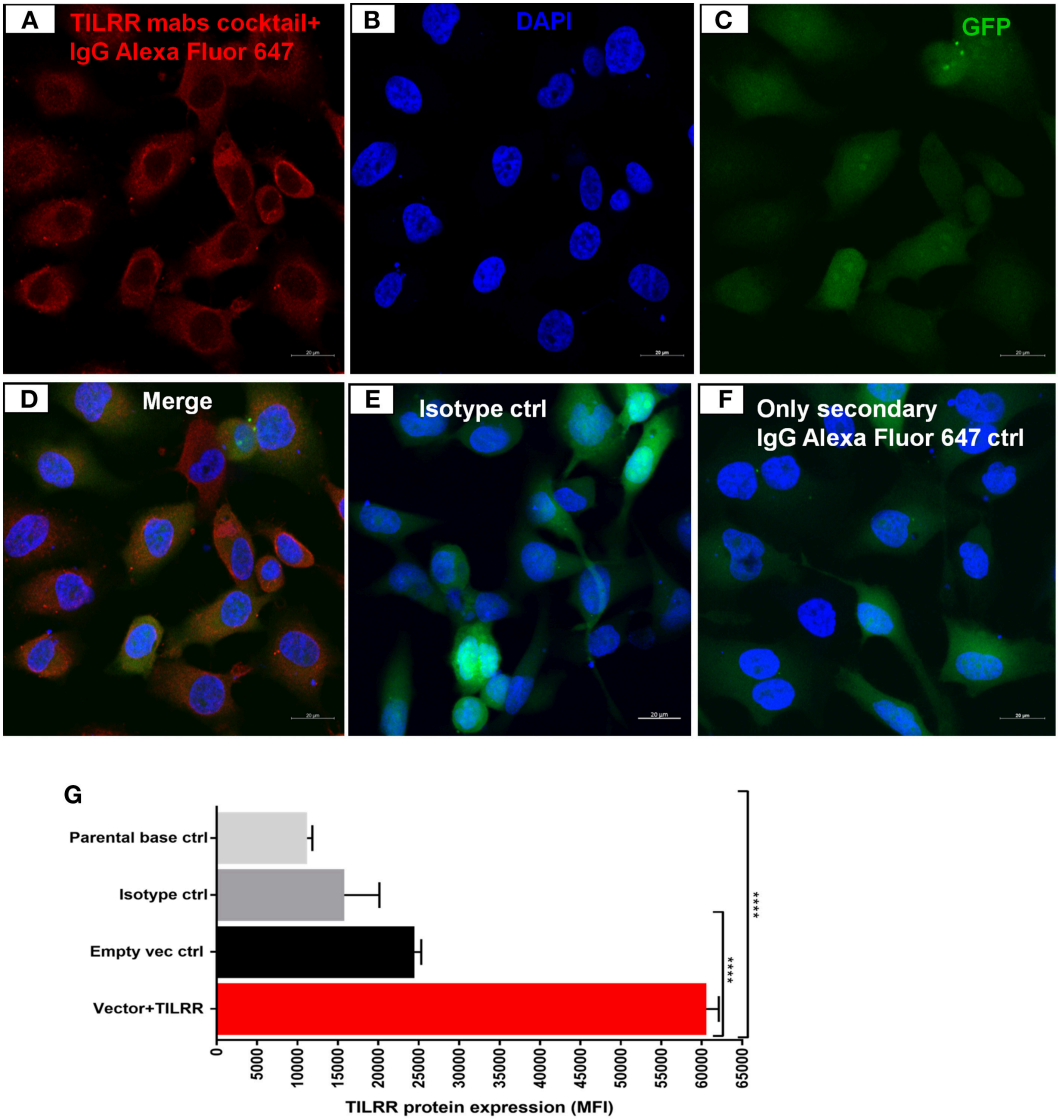
Confocal microscopy and flow cytometry analysis of TILRR protein overexpression in transfected cells. **(A)** TILRR protein expression in HeLa cells transfected with TILRR plasmid DNA. **(B)** DAPI staining shown cell nuclei. **(C)** Transfected cells showing eGFP. **(D)** Merged expression of TILRR protein, eGFP and nucleus **(E)** Isotype control monoclonal antibody (F400G3S) staining. **(F)** Alexa Fluor 647 labeled goat anti-mouse IgG secondary antibody only control. Color code: red, TILRR protein (Alexa Fluor 647 channel); blue, nuclear DNA (DAPI channel); and green, eGFP (FITC channel). Image captured using 20x objectives with 20 μm scale. **(G)** Flow cytometry confirmation of overexpressed TILRR protein in transfected HeLa cells. Cells were prepared by recommended protocol as illustrated in materials and methods section. The overexpressed TILRR protein confirmed by analyzing the mean fluorescence intensity (MFI) in TILRR-transfected cells compared to the parental base, isotype, and empty vector-transfected control cells. Student *t*-test with 95% CI performed for the statistical analysis using GraphPad prism version 7.03, all *p* < 0.05 were reported and indicated using an asterisks' ^****^p < 0.0001.

Western blot analysis of transfected cells showed that cells transfected with TILRR expressed significantly higher amount of TILRR protein compared to parental-HeLa cells and cells transfected with empty vector (*p* < 0.05) (Figure 2K; full length original blots presented in Figure S2). RT^2^ qPCR Primer analysis showed that cells transfected with TILRR containing plasmid significantly overexpressed the TILRR mRNA compared to non-transfected parental control and empty vector (*p* < 0.0001; Figure 2L). Confocal microscopy imaging analysis further confirmed the overexpressed TILRR protein in HeLa cells (Figures 3A–D and Figures S3A–D) compared to the respective controls (Figures 3E,F and Figures S3E–L). We also confirmed the TILRR protein expression in transfected HeLa cells by flow cytometry analysis using Alexa Fluor 647 labeled monoclonal antibodies (F218G1 and F218G5) recognizing epitopes in TILRR. The mean fluorescence intensity (MFI) of TILRR transfected cells showed significantly higher expression of TILRR compared to the parental control, isotype control and empty vector transfected control (Figure 3G, detailed gating strategy provided in Figure S4). These data indicated that TILRR transfected HeLa and VK2/E6E7 cells overexpressed TILRR.

### TILRR Overexpression Regulates the mRNA Transcript Expression of Inflammation Responsive Genes in a Dose Dependent Manner

**Figure 4:**
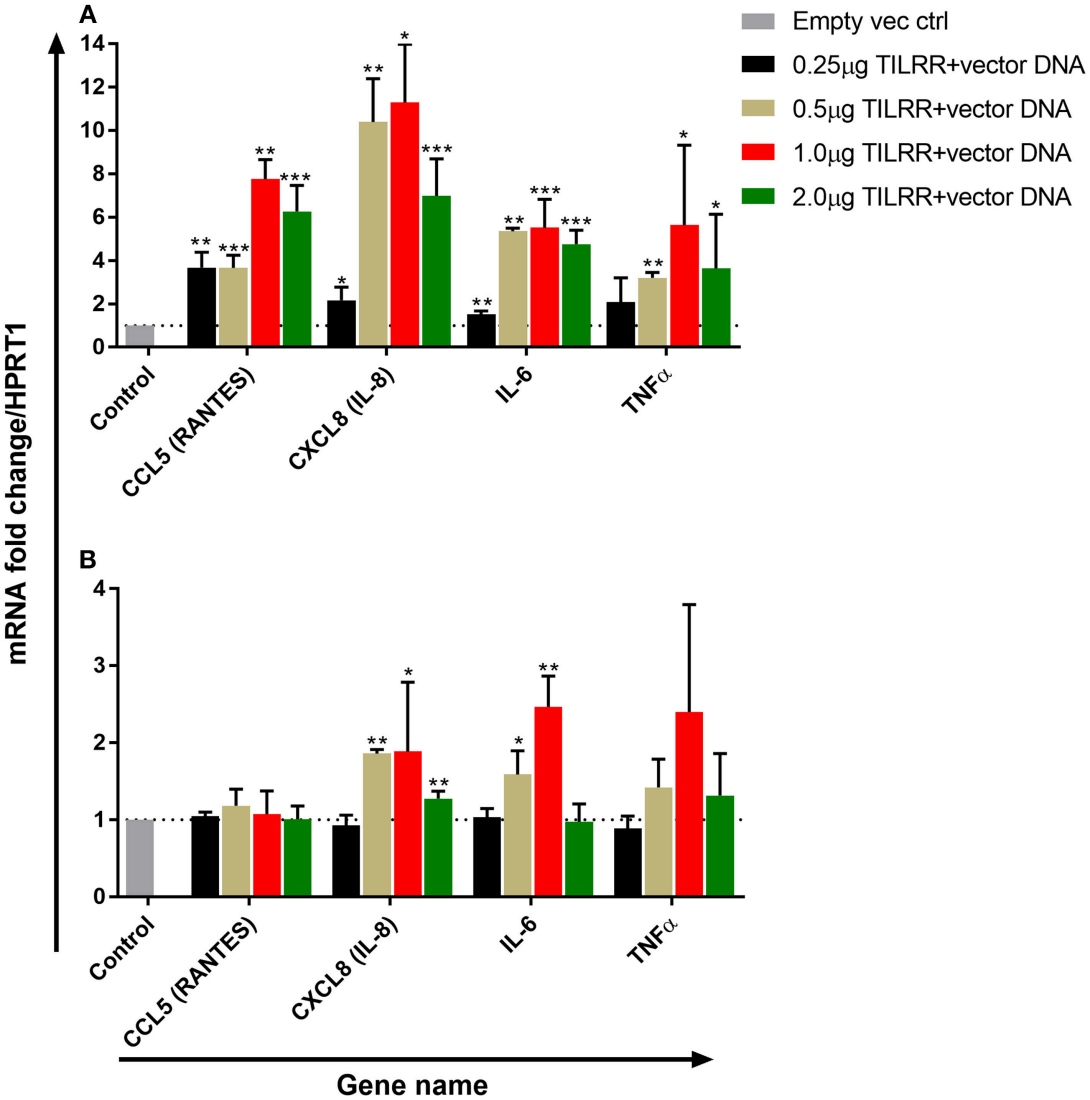
Dose response effect of TILRR overexpression on regulation of inflammation responsive genes: RT^2^ qPCR Primer assay for mRNA transcripts of the 4 immune and inflammation responsive genes in HeLa **(A)** and VK2/E6E7 **(B)** cells. Data were analyzed using GeneGlobe Data Analysis Centre (Qiagen) for RT^2^ qPCR Primer assay. Fold induction for individual gene in each concentration of TILRR-plasmid DNA transfected cells expressed as relative to levels of the respected concentration of empty vector transfected control and show mean ± SEM of three independent experiments. All data were normalized against HPRT1 housekeeping gene. The statistical comparisons conducted using student *t*-test, all *p* < 0.05 were reported and indicated using an asterisks' ^*^p < 0.05, ^**^p < 0.01, and ^***^p < 0.001. Legends on the upper right corner represent the experimental conditions with plasmid DNA concentrations.

To examine the effect of different amount of TILRR on immune and inflammation responsive genes, we co-transfected both HeLa and VK2/E6E7 cells with different concentration of TILRR (vector+TILRR) (0.25–2.0 μg/well) or empty vector control (0.25–2.0 μg/well) plasmid DNA in combination with PmaxGFP plasmid DNA (0.05–0.4 μg/well) and incubated at 37°C for 24 h as described in materials and methods section. We selected 4 genes (CCL5/RANTES, CXCL8/IL-8, IL-6, and TNFα) playing important role in immune activation and inflammatory response to evaluate the effect of different amount of TILRR on their mRNA expression. Different amount of TILRR-plasmid DNA transfection did result in a dose-dependent increase in mRNA expression in HeLa cells (Figure 4A) and in VK2/E6E7 cells (Figure 4B). HeLa cells transfected with 0.25–2.0 μg of TILRR-plasmid DNA significantly increased mRNA of all 4 genes in a dose dependent manner except TNFα, which was only significantly increased with the dose of 0.5 μg or above (Figure 4A; Table S1). In the case of VK2/E6E7 cells (Figure 4B), the effect of different amount of TILRR-plasmid DNA transfection was only observed for the mRNA of CXCL8 and IL-6 (Figure 4B and Table S2). The data showed that one microgram of plasmid DNA worked best in upregulating the selected inflammation responsive genes (GeneCopoeia recommended protocol). Thus, we used 1.0 μg of plasmid DNA for all subsequent experiments in this study.

### Overexpression of TILRR Significantly Influenced the mRNA Level of Genes in NFκB Signal Transduction Pathway

Next, we investigated the effect of TILRR overexpression on the 84 genes in the NFκB signal transduction pathway. We hypothesized that upon interaction with IL-1R1 receptor, TILRR would influence the downstream signaling events by regulating mRNA transcript of genes in the NFκB signal transduction pathway. To test this, we investigated the effect of overexpression of TILRR on a panel of 84-genes (Tables S3, S4) that are directly related to NFκB signaling pathway, immune activation and inflammatory responses using the Human NFκB pathway RT^2^ profiler PCR array.

**Figure 5:**
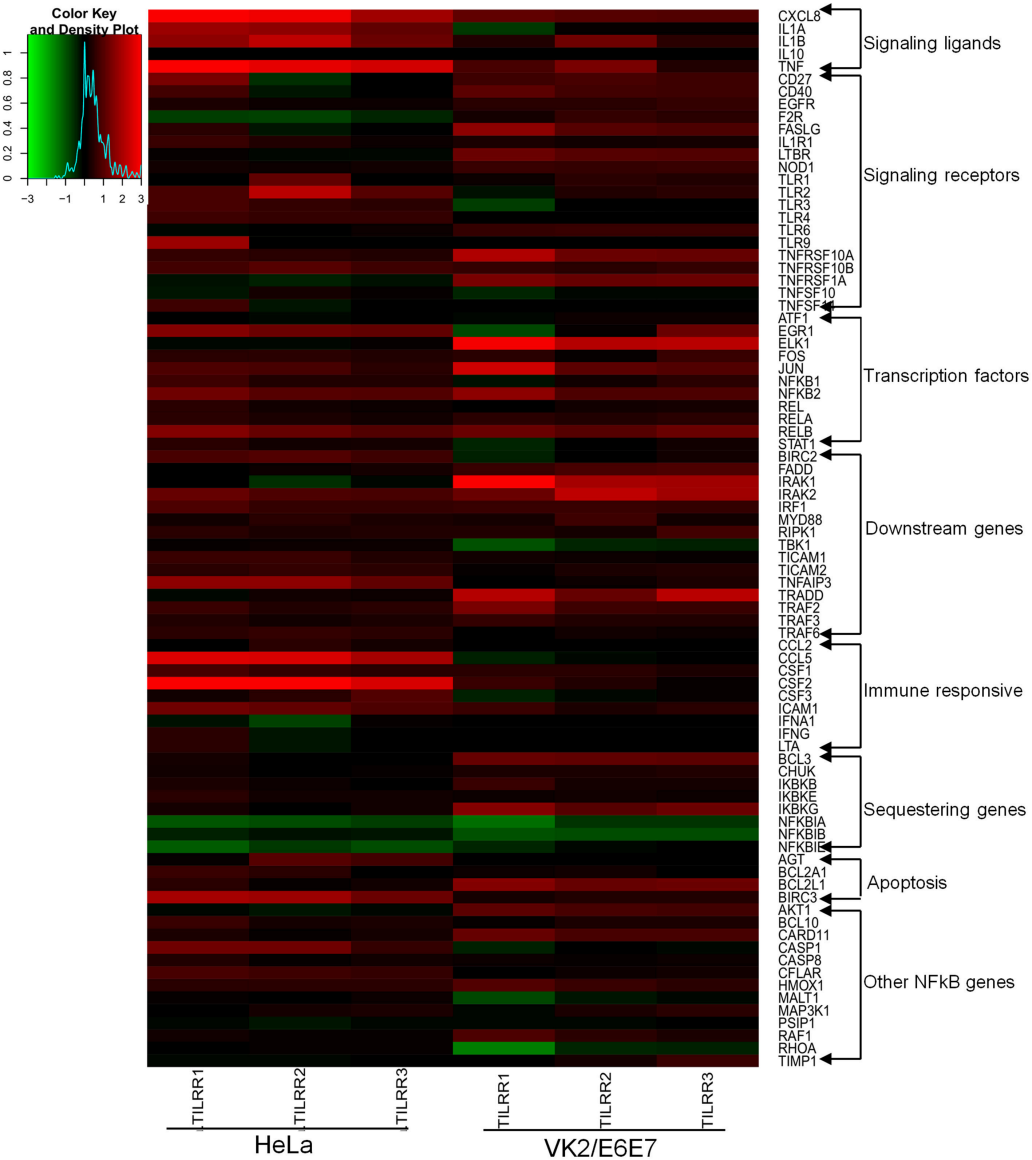
Heat map presentation of up- and down- regulated genes in NFκB pathway for the presence of TILRR. Log2 fold changes of gene expression in HeLa and VK2/E6E7 cells generated by RStudio (https://www.rstudio.com). Gradient red color indicates the up-regulated genes; gradient green represents the down-regulated genes. Solid black means baseline, which represents the fold change 1 or log2 = 0 (control). “X” axis showing the triplicate biological samples for each cell line, “Y” axis (right side) represents the 84 tested genes categorized into different groups. Legend on the upper left side shows the scale of log2 fold change.

**Figure 6:**
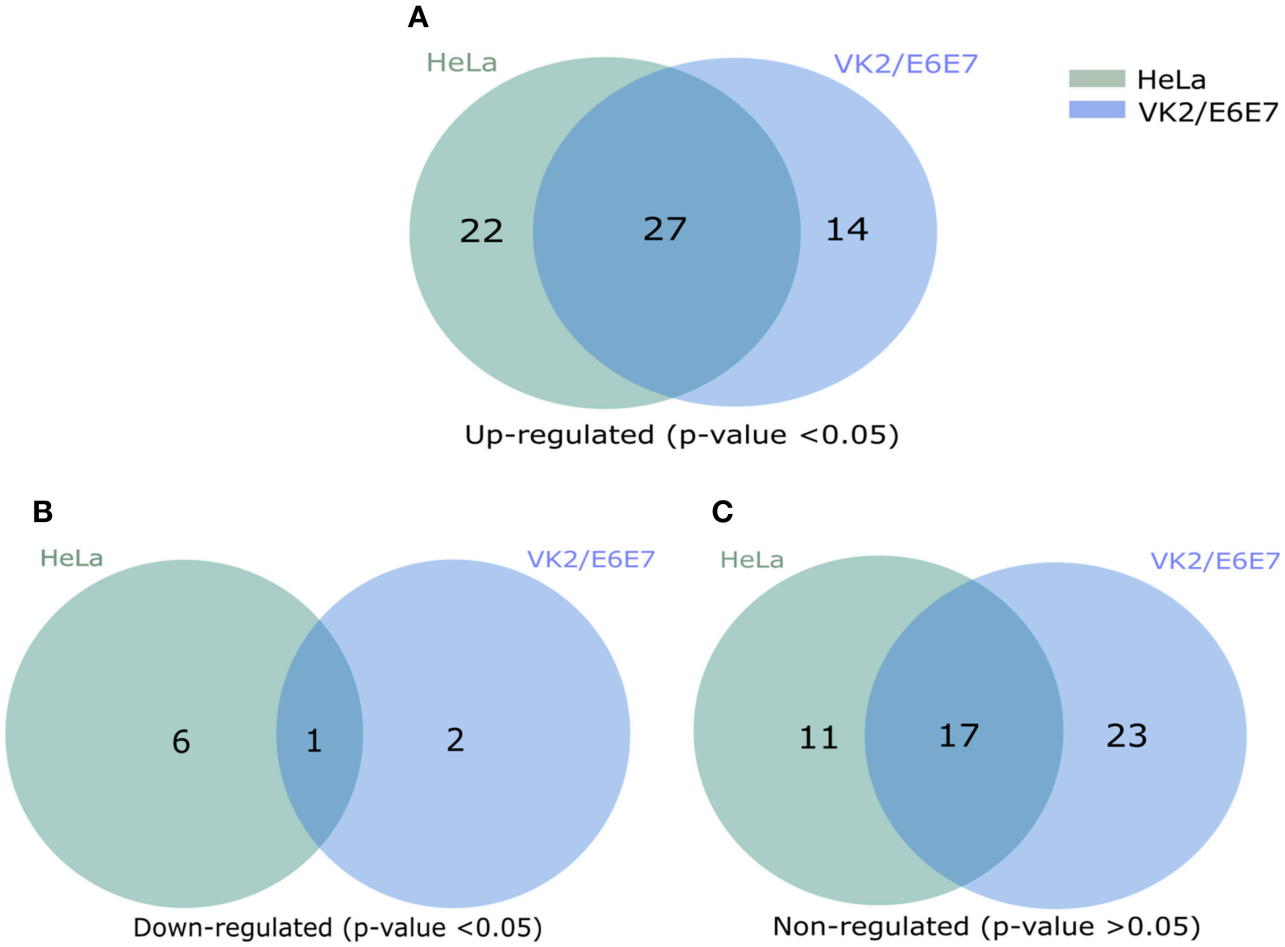
Venn diagram presentation of total regulated genes in NFκB signal transduction pathway. **(A)** The significantly up-regulated genes in HeLa and VK2/E6E7 cells (*p* < 0.05). **(B)** Significantly down-regulated genes in both cell lines (*p* < 0.05). **(C)** Non-influenced genes in both HeLa and VK2/E6E87 for the presence of TILRR (*p* > 0.05). Legends on the upper right corner represent the cell lines. Right circle represents the HeLa cells, left circle for VK2/E6E7 cells, and centre overlapping circle represents the combined regulated genes in both cell lines. Venn diagrams were made using pyvenn (https://github.com/tctianchi/pyvenn).

We first assessed the mRNA transcript fold change of NFκB signal transduction related genes in transfected HeLa cells after 3 h of incubation with serum free DMEM. The result showed fold increase (FI) in mRNA expression of a few genes were significantly upregulated (CCL5, FI: CXCL8, FI: 3.08 ± 0.78, *p* = 0.0026; IRAK2, FI: 1.30 ± 0.13, *p* = 0.0256; IRAF1, FI: 1.26 ± 0.18, *p* = 0.0316; RIPK1, FI: 1.08 ± 0.03, *p* = 0.0417, TICAM1, FI: 1.13 ± 0.08, *p* = 0.0273; TLR4, FI: 1.14 ± 0.07, *p* = 0.0113) by TILRR overexpression at the 3 h time point (Figures S5A–F). The mRNA expressions of more genes were significantly influenced by the overexpression of TILRR in both cell lines at 24 h as presented in the heat map (Figure 5). Of the 84 genes, 49- and 41-genes were significantly up-regulated in HeLa and VK2/E6E7 cells, respectively (Figure 6A). Seven genes were significantly down-regulated in HeLa (AKT1, FI: 0.87 ± 0.09, *p* = 0.0277; F2R, FI: 0.64 ± 0.20, *p* = 0.0046; NFKBIA, FI: 0.54 ± 0.06, *p* = 0.0011; NFKBIB, FI: 0.80 ± 0.04, *p* = 0.0020; NFKBIE, FI: 0.54 ± 0.09, *p* = 0.0015; PSIP1, FI: 0.87 ± 0.08, *p* = 0.0098; TNFRSF1A, FI: 0.81 ± 0.06, *p* = 0.0055); 3 genes were down-regulated in VK2/E6E7 cells (NFKBIB, FI: 0.53 ± 0.01, *p* = 0.0057; STAT1, FI: 0.55 ± 0.16, *p* = 0.0442; and TBK1, FI: 0.67 ± 0.14, *p* = 0.0309) (Figure 6B). No significant fold change observed for 28 and 40 genes in HeLa and VK2/E6E7 cells, respectively (Figure 6C).

**Figure 7:**
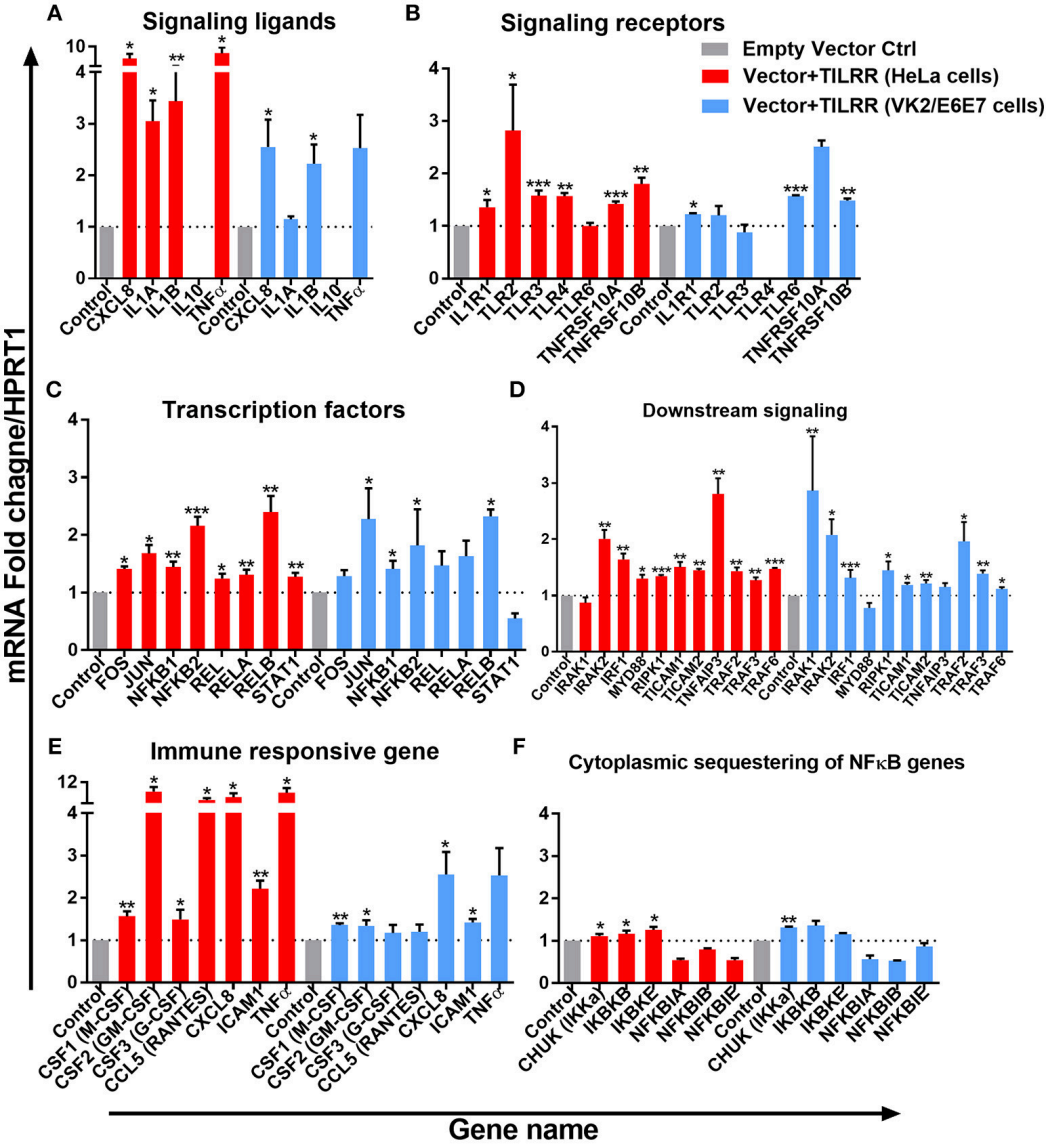
TILRR induces expression of genes in NFκB signal transduction pathway. HeLa and VK2/E6E7 cells were transiently co-transfected with either pEZ-TILRR-M68 (vector+TILRR) (1.0 μg/5 × 10^5^ cells) or pEZ-NEG-M68 (empty vector control) (1.0 μg/5 × 10^5^ cells) along with PmaxGFP (0.2 μg/5 × 10^5^ cells) in parallel experiments for 24 h at 37°C with 5% CO_2_ as described in material and methods section. Harvested RNAs from both cells used for synthesis of cDNAs that finally run with RT^2^ profiler qPCR array targeting NFκB signaling components such as signaling ligands **(A)**, signaling receptors **(B)**, transcription factors **(C)**, downstream signaling genes **(D)**, immune responsive genes **(E)**, and cytoplasmic sequestering of NFκB genes **(F)**. Data were analyzed using GeneGlobe Data Analysis Centre (Qiagen) for RT^2^ profiler PCR array. Fold induction for individual gene in TILRR transfected cells expressed as relative to levels of empty vector transfected control and show mean ± SEM of three independent experiments. All data were normalized against HPRT1 housekeeping gene. The statistical comparisons conducted using student *t*-test, all *p* < 0.05 were reported and indicated using an asterisks' ^*^p < 0.05, ^**^p < 0.01, and ^***^p < 0.001. Legends on the upper right corner represent the cells with experimental conditions.

The expression of NFκB signaling ligands, such as CXCL8 (IL-8) and IL-1β were significantly up-regulated in HeLa cells (CXCL8, FI: 6.33 ± 2.48, *p* = 0.0154; and IL-1β, FI: 3.44 ± 1.15, *p* = 0.0084) and in VK2/E6E7 cells (CXCL8, FI: 2.55 ± 0.92, *p* = 0.0252; IL-1β, FI: 2.23 ± 0.64, *p* = 0.0379). The magnitude of increase of these signaling ligands is higher in HeLa cells. Significant increases in IL-1α (FI: 3.05 ± 0.70, *p* = 0.0126) and TNFα (FI: 5.63 ± 3.69, *p* = 0.0100) expression were only observed in HeLa cells. Overexpression of TILRR appears to have no effect on mRNA of IL-10 detection in these two cell lines (Figure 7A).

Overexpressing TILRR in HeLa cells significantly increased the expression of 6 out of 7-signaling receptors including IL-1R1 (FI: 1.36 ± 0.24, *p* = 0.0263), TLR2 (FI: 2.82 ± 1.52, *p* = 0.0278), TLR3 (FI: 1.58 ± 0.17, *p* = 0.0004), TLR4 (FI: 1.57 ± 0.10, *p* = 0.0055), TNFRSF10A (FI: 1.42 ± 0.08, *p* = 0.0003) and TNFRSF10B (FI: 1.80 ± 0.21, *p* = 0.0012) (Figure 7B). In particular, IL-1R1 and TNFRSF10B receptor expression were significantly up-regulated in both HeLa (IL-1R1, FI: 1.36 ± 0.24, *p* = 0.0263 and TNFRSF10B, FI: 1.80 ± 0.21, *p* = 0.0012) and VK2/E6E7 (IL-1R1, FI: 1.23 ± 0.03, *p* = 0.0238 and TNFRSF10B, FI: 1.49 ± 0.06, *p* = 0.0032) cells, respectively. However, TLR6 (FI: 1.57 ± 0.03, *p* = 0.0007) expression was only significantly increased in VK2/E6E7 cells, and TLR6 mRNA was unaltered in TILRR transfected HeLa cells. Further, there was no significant effect on TLR4 mRNA detection in VK2/E6E7 cells.

TILRR overexpression enhanced the expression of 8 NFκB transcription factors (Figure 7C) in HeLa cells. Of which, the expression of FOS (FI: 1.41 ± 0.07, *p* = 0.0124), JUN (FI: 1.68 ± 0.25, *p* = 0.0266), NFκB1 (p105/p50) (FI: 1.44 ± 0.16, *p* = 0.0036), NFκB2 (p100/p52) (FI: 2.16 ± 0.27, *p* = 0.0004), REL (p65) (FI: 1.25 ± 0.14, *p* = 0.0182), RELA (FI: 1.31 ± 0.15, *p* = 0.0091), RELB (FI: 2.40 ± 0.48, *p* = 0.0066), and STAT1 (FI: 1.27 ± 0.12, *p* = 0.0046) was significantly increased. However, only the expression of four NFκB transcription factors significantly increased in VK2/E6E7 cells, including JUN (FI: 2.28 ± 0.92, *p* = 0.0499), NFκB1 (p105/p50) (FI: 1.41 ± 0.24, *p* = 0.0421), NFκB2 (p100/p52) (FI: 1.82 ± 1.09, *p* = 0.0494) and RELB (FI: 2.32 ± 0.21, *p* = 0.0173).

The expression of mRNA of genes directly connected to the downstream NFκB signaling was significantly increased following TILRR overexpression in both cell lines (Figure 7D). These included IRAK2 (FI: 2.01 ± 0.27, *p* = 0.0024 and FI: 2.08 ± 0.48, *p* = 0.0182), IRF1 (FI: 1.64 ± 0.18, *p* = 0.0043 and FI: 1.32 ± 0.24, *p* = 0.0002), RIPK1 (FI: 1.34 ± 0.04, *p* = 0.0002, and FI: 1.45 ± 0.27, *p* = 0.0361), TICAM1 (FI: 1.51 ± 0.15, *p* = 0.0030, and FI: 1.19 ± 0.06, *p* = 0.0247), TICAM2 (FI: 1.45 ± 0.05, *p* = 0.0093, and FI: 1.21 ± 0.11, *p* = 0.0093), TRAF2 (FI: 1.43 ± 0.11, *p* = 0.0023, and FI: 1.96 ± 0.59, *p* = 0.0319), TRAF3 (FI: 1.27 ± 0.09, *p* = 0.0080, and FI: 1.39 ± 0.09, *p* = 0.0061) and TRAF6 (FI: 1.47 ± 0.03, *p* = 0.0004, and FI: 1.12 ± 0.06, *p* = 0.0247) in HeLa and VK2/E6E7 cells, respectively. In addition, MYD88 (FI: 1.30 ± 0.12, *p* = 0.0425) and TNFAIP3 (FI: 2.81 ± 0.48, *p* = 0.0049) were also enhanced in HeLa cells, whereas IRAK1 (FI: 2.86 ± 1.67, *p* = 0.0013) in VK2/E6E7 cells only.

The expression of immune responsive genes directly associated with the NFκB signaling pathway was also examined. We observed significant induction of mRNA transcripts for 7 immuno-regulatory genes when TILRR was overexpressed (Figure 7E). The expression of CSF1 (M-CSF) (FI: 1.56 ± 0.20, *p* = 0.0065 and FI: 1.35 ± 0.08, *p* = 0.0031), CSF2 (GM-CSF) (FI: 8.46 ± 2.93, *p* = 0.0261 and FI: 1.34 ± 0.23, *p* = 0.0249), CXCL8 (IL-8) (FI: 6.33 ± 2.48, *p* = 0.0154, and FI: 2.55 ± 0.92, *p* = 0.0252), and ICAM1 (FI: 2.22 ± 0.33, *p* = 0.0011 and FI: 1.42 ± 0.14, *p* = 0.0155) was enhanced in TILRR transfected HeLa and VK2/E6E7 cells, respectively. CSF3 (G-CSF) and CCL5 (RANTES) were only significantly regulated in HeLa cells.

Finally, we examined the effect of TILRR on the expression of genes that are involved in cytoplasmic sequestration or release of NFκB complex proteins (Figure 7F). The expression of three out of six genes evaluated was significantly up-regulated in HeLa cell line, including CHUK (IKKa), IKBKB (IKKβ), and IKBKE (IKKε). Only the expression of CHUK was up-regulated in VK2/E6E7 cells. Thus, TILRR has direct influence on genes involved in formation of transcription factors NFκB1 (p50) and NFκB2 (p52) that subsequently translocate to the nucleus and potentiate signal transduction.

**Figure 8:**
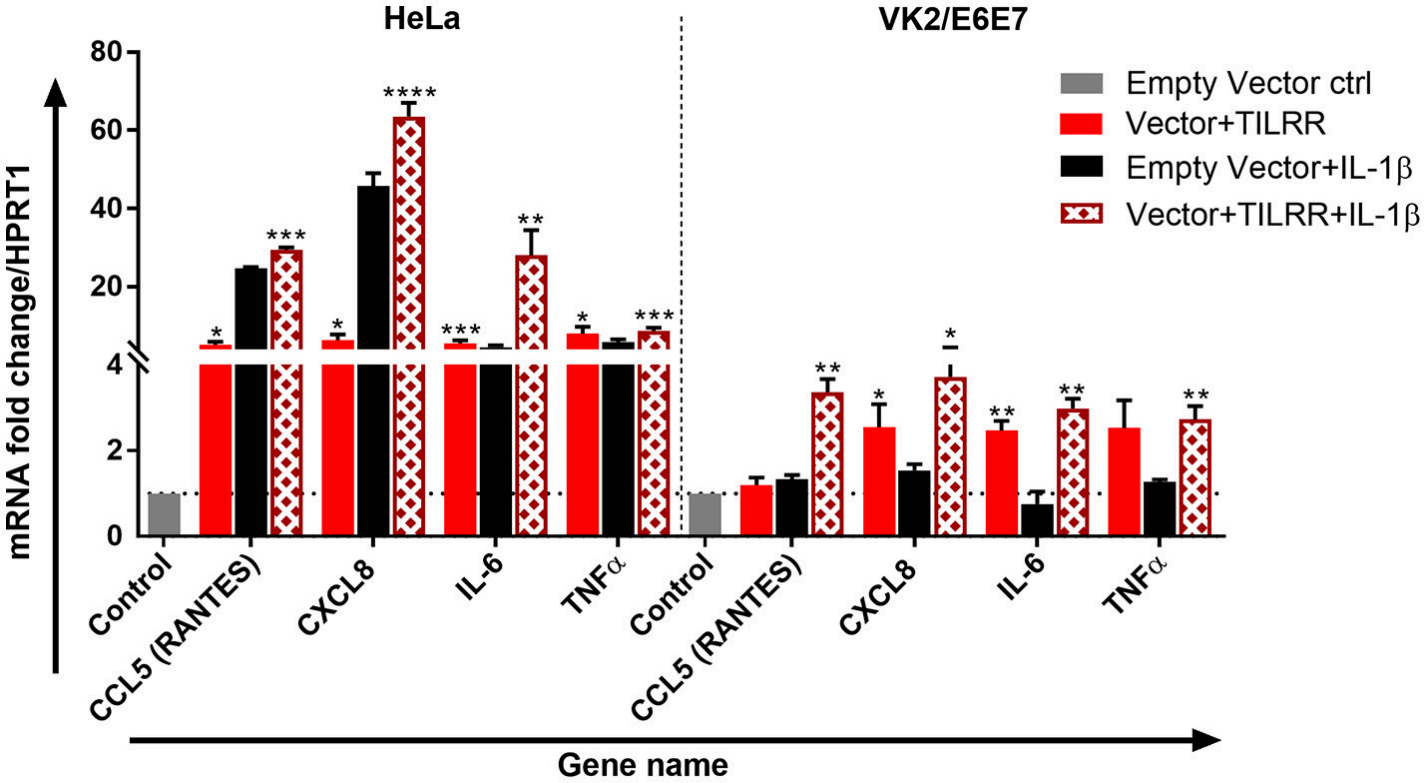
TILRR enhances the expression of immune responsive genes in the presence or absence of added IL-1β. HeLa (5 × 10^5^/well) and VK2/E6E7 (5 × 10^5^/well) cells were transiently co-transfected with either pEZ-TILRR-M68 (vector+TILRR) (1.0 μg/well) or pEZ-NEG-M68 (empty vector control) (1.0 μg/well) along with PmaxGFP (0.2 μg/well) in parallel experiments as described in materials and methods. In parallel experiments, the cells were incubated with the addition of IL-1β (1 nM) in serum free DMEM (HeLa) or Keratinocyte SFM (1X) (VK2/E6E7) media for 24 h at 37°C. Data were analyzed using GeneGlobe Data Analysis Centre (Qiagen) for RT^2^ qPCR Primer assay. Fold increase for individual gene in TILRR transfected (vector+TILRR) cells, in the presence or absence of IL-1β, expressed as relative to levels of empty vector transfected control and show mean ± SEM of three independent experiments. All data were normalized against HPRT1 housekeeping gene. The statistical comparisons conducted using student *t*-test, all *p* < 0.05 were reported and indicated using an asterisks' ^*^p < 0.05, ^**^p < 0.01, ^***^p < 0.001, and ^****^p < 0.0001. Legends on the upper right corner represent the cells with experimental conditions.

We further tested the effect of TILRR overexpression in the presence of IL-1β on selected genes directly involved in immune activation and inflammatory response in HeLa and VK2/E6E7 cells. TILRR transfected HeLa and VK2/E6E7 cells were incubated with or without IL-1β in parallel experiments and the expression of mRNA transcript was quantified using RT^2^ qPCR Primer assay. The results showed that TILRR overexpression, in the presence or absence of added IL-1β, significantly increased the expression of 4 immune and inflammation responsive genes in HeLa and VK2/E6E7 cells (Figure 8).

In the absence of added IL-1β, TILRR overexpression significantly increased mRNA transcript fold change of CXCL8 (IL-8) (FI: 6.33 ± 2.48, *p* = 0.0154), IL-6 (FI: 5.52 ± 1.30, *p* = 0.005), and TNFα (FI: 5.63 ± 3.69, *p* = 0.0100) in HeLa cells; and CXCL8 (IL-8) (FI: 2.55 ± 0.92, *p* = 0.0252) and IL-6 (FI: 2.47 ± 0.40, *p* = 0.0031) in VK2/E6E7 cells compared to empty vector control. CCL5 (RANTES) (FI: 5.24 ± 1.23, *p* = 0.0116) was an exception, which was only significantly enhanced in HeLa cells for the TILRR overexpression. In the presence of added IL-1β, TILRR overexpression also significantly increased mRNA transcripts of these immune and inflammation responsive genes in comparison to the empty control vector. These include CCL5 (RANTES) (FI: 27.48 ± 3.25, *p* = 0.0006), CXCL8 (IL-8) (FI: 63.58 ± 5.94, *p* < 0.0001), IL-6 (FI: 28.03 ± 11.08, *p* = 0.0057), and TNFα (FI: 8.67 ± 1.53, *p* = 0.0005) in HeLa cells; and CCL5 (RANTES) (FI: 3.66 ± 0.54, *p* = 0.0016), CXCL8 (IL-8) (FI: 3.72 ± 1.23, *p* = 0.0183), IL-6 (2.98 ± 0.40, *p* = 0.0010), and TNFα (FI: 2.73 ± 0.54, *p* = 0.0051) in VK2/E6E7 cells. These results support that TILRR acts as a co-receptor of IL-1R1, which enhances the activation of NFκB signaling genes in the inflammatory response during microbial infection.

### TILRR Overexpression Significantly Increased the Production of Pro-Inflammatory Cytokine/Chemokine Proteins

**Figure 9:**
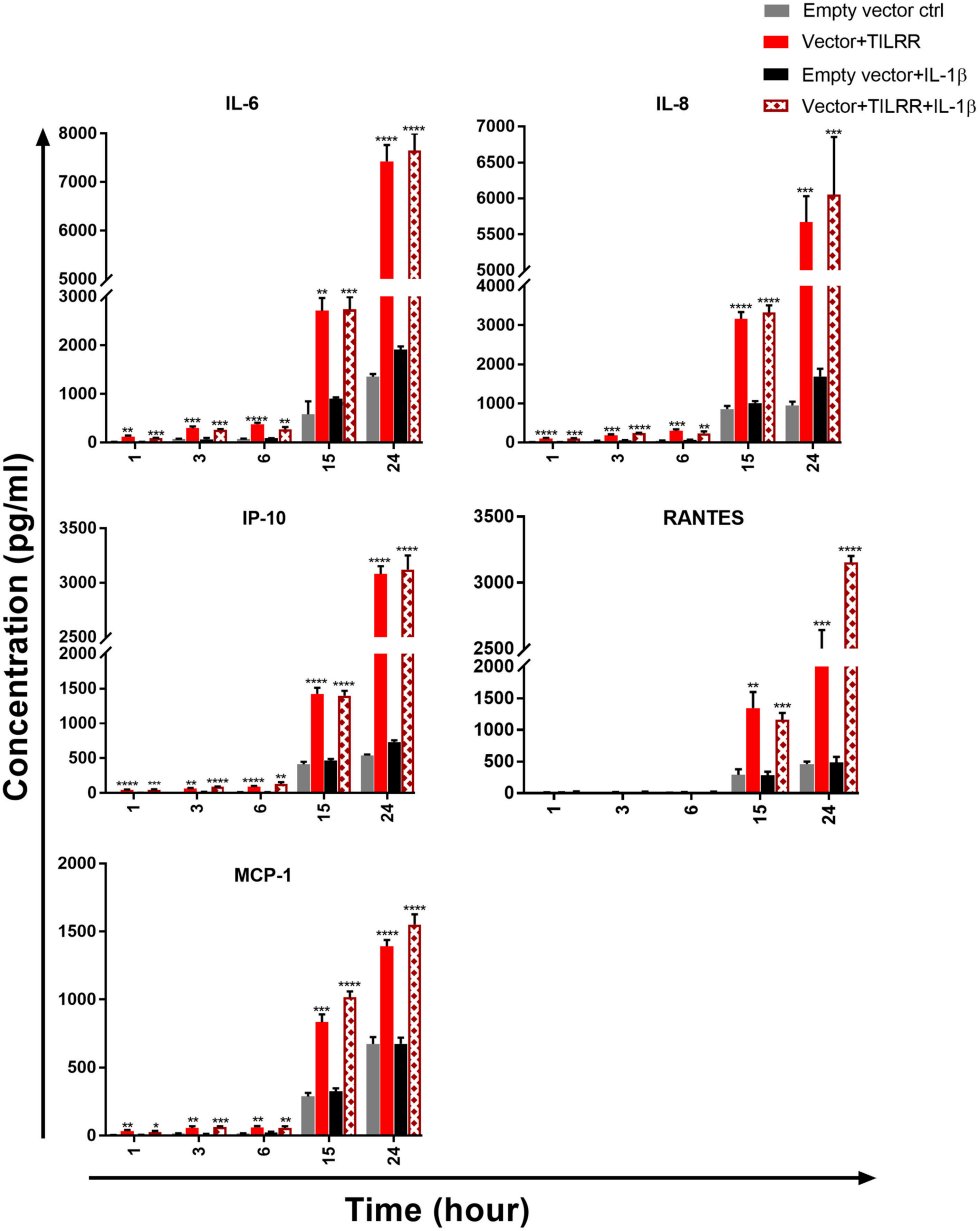
TILRR overexpression in HeLa cells increased the production of Pro-inflammatory cytokine/chemokine(s) in the presence or absence of added IL-1β. HeLa (5 × 10^5^cells/well) cells were co-transfected with either pEZ-TILRR-M68 (1.0 μg/5 × 10^5^ cells) or pEZ-NEG-M68 (1.0 μg/5 × 10^5^ cells) with PmaxGFP (0.2 μg/5 × 10^5^ cells) vector as explained in materials and methods section. In parallel, cells were incubated with or without the addition of IL-1β (1 nM) in serum free DMEM (HeLa) media and then the cultured media were collected (see methods). Thirteen different inflammatory cytokines were measured using an in-house developed multiplex cytokine/chemokine(s) bead assay with BioPlex 200 (BIORAD). The data represent the relative level of vector + TILRR, in the presence or absence of IL-1β, compared to the empty vector control. The sample measurements below the detection limit were assigned as zero. The data represent mean of three independent experiments (mean ± SEM). Statistical comparisons conducted using student *t*-test with 95% CI, all *p* < 0.05 were reported and indicated using an asterisks' ^*^p < 0.05, ^**^p < 0.01, ^***^p < 0.001, and ^****^p < 0.0001.

**Figure 10:**
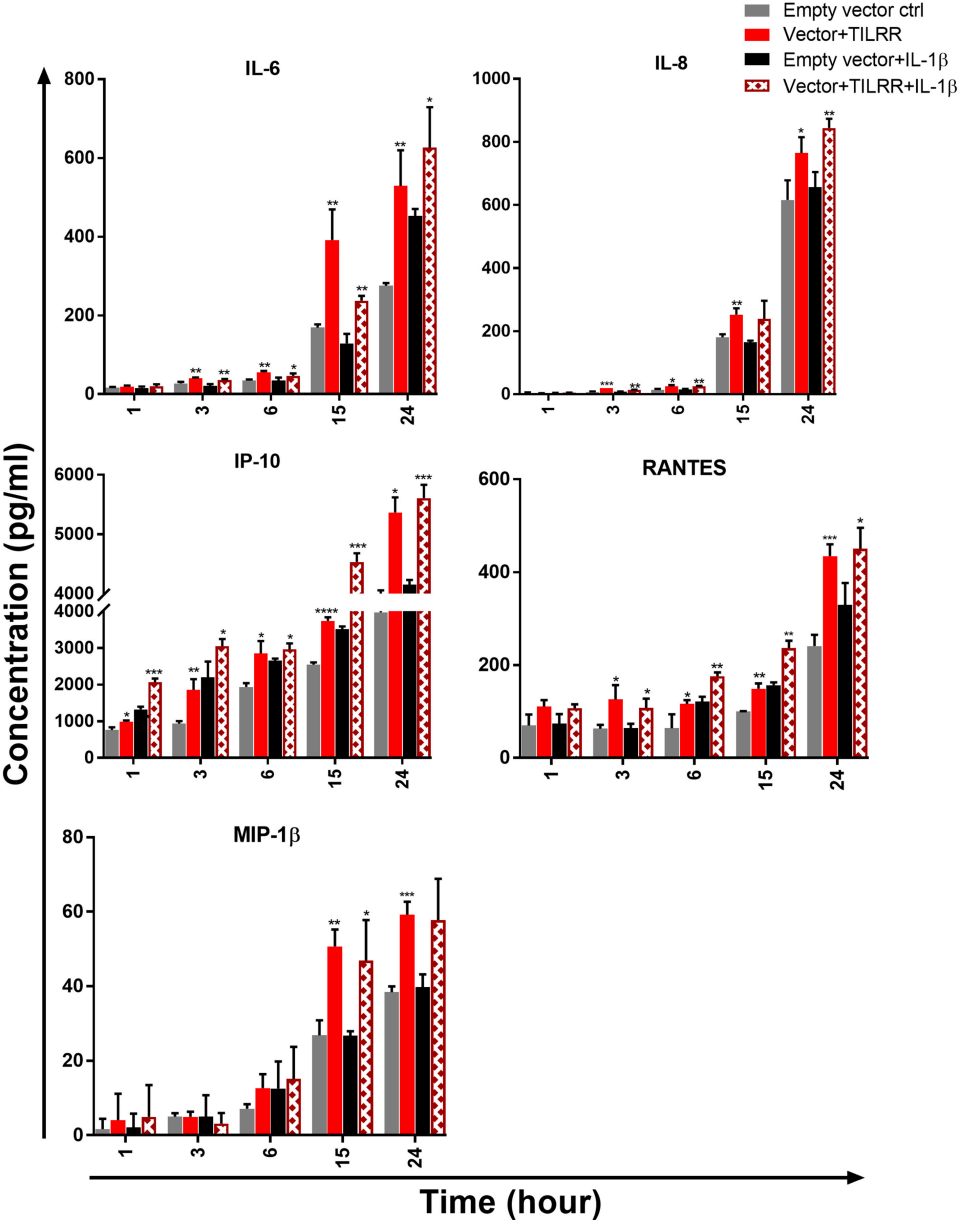
TILRR overexpression in VK2/E6E7 cells increased the production of Pro-inflammatory cytokine/chemokine(s) in the presence or absence of added IL-1β. VK2/E6E7 (5 × 10^5^cells/well) cells were co-transfected with either pEZ-TILRR-M68 (1.0 μg/5 × 10^5^ cells) or pEZ-NEG-M68 (1.0 μg/5 × 10^5^ cells) with PmaxGFP (0.2 μg/5 × 10^5^ cells) vector as explained in materials and methods section. In parallel, cells were incubated with or without the addition of IL-1β (1 nM) in serum free Keratinocyte SFM (1X) (VK2/E6E7) media and then the cultured media were collected (see methods). Thirteen different inflammatory cytokines were measured using an in-house developed multiplex cytokine/chemokine(s) bead assay with BioPlex 200 (BIORAD). The data represent the relative level of vector+TILRR, in the presence or absence of IL-1β, compared to the empty vector control. The sample measurements below the detection limit were assigned as zero. The data represent mean of three independent experiments (mean ± SEM). Statistical comparisons conducted using student *t-*test with 95% CI, all *p* < 0.05 were reported and indicated using an asterisks' ^*^p < 0.05, ^**^p < 0.01, ^***^p < 0.001, and ^****^p < 0.0001.

To examine whether the production of specific cytokine/chemokine of TILRR transfected cells was also influenced by the overexpression of TILRR, we quantified the production of 13 cytokine/chemokine(s) (Table S5) in TILRR transfected HeLa and VK2/E6E7 cell culture supernatants using multiplex bead assay. The results showed that TILRR overexpression, in the presence or absence of added IL-1β, significantly increased the production of several pro-inflammatory cytokine/chemokines in HeLa (Figure 9) and VK2/E6E7 cells (Figure 10) compared to the empty vector control during the observed time points from 1 to 24 h. In the absence of IL-1β, TILRR overexpression significantly increased the cytokine/chemokine(s) production in HeLa cell culture supernatants at different time points (Figure 9). After 1 h incubation with serum free media, IL-6 (*p* = 0.0014), IL-8 (CXCL8) (*p* < 0.0001), IP-10 (*p* < 0.0001), and MCP-1 (*p* = 0.0064) were significantly increased in culture supernatants. After 3-, 6- and 15-h incubation, we observed significantly higher production of IL-6 (*p* = 0.0004, < 0.0001, and 0.0011), IL-8 (CXCL8) (*p* = 0.0004, 0.0005, and < 0.0001), IP-10 (*p* = 0.0012, < 0.0001, and < 0.0001), and MCP-1 (*p* = 0.0035, 0.0013, and 0.0001) in HeLa cell culture supernatants, except RANTES (CCL5) (*p* = 0.0024), which was only increased after 15h incubation. The level of IL-6 (*p* < 0.0001), IL-8 (CXCL8) (*p* = 0.0002), IP-10 (*p* < 0.0001), RANTES (CCL5) (*p* = 0.0001), and MCP-1 (*p* < 0.0001) in the cell culture supernatant of TILRR-transfected HeLa cells remained high at 24 h. The data showed the consistency between mRNA and protein level expression of IL-8 (CXCL8) and RANTES (CCL5). However, CSF2 (GM-CSF), IFNγ, IL-10, IL17A, MIP-1α, and TNFα were not detected in the HeLa cell culture supernatant (data not shown).

We next examined the level of cytokine/chemokine(s) production in culture supernatants of TILRR-overexpressed VK2/E6E7 cells. Similar to the HeLa cells, in the absence of IL-1β, there was a gradual increase of cytokine/chemokine(s) production in VK2/E6E7 cell culture supernatants at different time points compared to the empty vector-transfected cells (Figure 10). Unlike the HeLa cells, we observed that after 1 h incubation with serum free media, only IP-10 (*p* = 0.0109) was significantly increased in TILRR overexpressed VK2/E6E7 cell supernatants. However, after longer incubations time (3-, 6-, 15-, and 24-h incubation), the levels of IL-6 (*p* = 0.0061, 0.0013, 0.0077, and 0.0084), IL-8 (CXCL8) (*p* = 0.0007, 0.0243, 0.0050, and 0.0319), IP-10 (*p* = 0.0061, 0.0109, < 0.0001, and = 0.0301), and RANTES (CCL5) (*p* = 0.0283, 0.0435, 0.0016, and 0.0007) were all significantly increased. Whereas, the level of MIP-1β (*p* = 0.0025, and 0.0007) was only significantly increased after 15 h and 24 h incubation. CSF2 (GM-CSF), IFNγ, IL-10, IL17A, MCP-1, MIP-1α, and TNFα were not detected in the VK2/E6E7 cell culture supernatant after these incubation time (data not shown). Thus, overexpression of TILRR enhanced the production of many pro-inflammatory cytokine/chemokine(s) in culture supernatants.

To determine whether TILRR overexpression augments the production of pro-inflammatory cytokine/chemokine(s) in the presence of IL-1β, we analyzed the effect of TILRR in the presence of IL-1β in cell culture supernatants of HeLa and VK2/E6E7 cells. The analysis showed that in HeLa cells the level of these cytokine/chemokine(s) increased in a time-dependent manner with the presence of TILRR and added IL-1β in comparison to the empty vector-transfected control (Figure 9). After 1 h incubation in serum free media, the level of IL-6 (*p* = 0.0002), IL-8 (CXCL8) (*p* = 0.0001), IP-10 (*p* = 0.0002), and MCP-1 (*p* = 0.0368) was significantly increased in HeLa cells. After longer incubation times (3-, 6-, 15-, and 24-h incubation), the level of IL-6 (*p* = 0.0005, 0.0029, 0.0002, and < 0.0001), IL-8 (CXCL8) (*p* < 0.0001, = 0.0042, < 0.0001, and = 0.0008), IP-10 (*p* < 0.0001, = 0.0014, < 0.0001, and < 0.0001), and MCP-1 (*p* = 0.0001, 0.0088, < 0.0001, and < 0.0001) was significantly increased due to the combined effect of TILRR and IL-1β. The chemokine RANTES (CCL5) was only significantly increased at 15 h (*p* = 0.0002) and 24 h (*p* < 0.0001) after incubation with IL-1β in serum free media. CSF2 (GM-CSF), IFNγ, IL-10, IL17A, MIP-1α, MIP-1β, and TNFα were not detected in the HeLa cell culture supernatant with TILRR overexpression and added IL-1β (data not shown).

In the case of VK2/E6E7 cells, we observed a trend of increase of pro-inflammatory cytokine/chemokine(s) production in the presence of TILRR and IL-1β compared to the empty vector control in serum free media (Figure 10). Unlike the HeLa cells, only IP-10 (*p* = 0.0004) was significantly higher with the TILRR overexpression and added IL-1β in VK2/E6E7 cell supernatants after 1 h incubation. Similar to HeLa cells, after longer incubation time (3- and 24-h incubation) the levels of IL-6 (*p* = 0.0058 and 0.0439), IL-8 (CXCL8) (*p* = 0.0011 and 0.0045), IP-10 (*p* = 0.0362 and 0.0004) and RANTES (*p* = 0.0281 and 0.0329) were significantly increased in VK2/E6E7 cell culture supernatants. The significant increase of IL-8 (CXCL8) (*p* = 0.0049), IP-10 (*p* = 0.0384) and RANTES (*p* = 0.0021) was also observed after 6h incubation and the higher level of IL-6 (*p* = 0.0022), IP-10 (*p* = 0.0004), RANTES (CCL5) (*p* = 0.0012) and MIP-1β (*p* = 0.0332) was also observed after 15h incubation. However, the production of CSF2 (GM-CSF), IFNγ, IL-10, IL17A, MCP-1, MIP-1α, and TNFα was not detected following addition of IL-1β in the VK2/E6E7 cell culture supernatant (data not shown). Altogether, these data suggested that TILRR, in the presence or absence of added IL-1β, can modulate the production of pro-inflammatory cytokine/chemokines during inflammatory process and microbial infection.

## Discussion

Previous studies identified a variant of FREM1 as a co-receptor of IL-1R1, and its association with IL-1R1 enhances the recruitment of MYD88, controls the induction of Ras GTPase and amplifies the activation of NFκB and inflammatory responses (18, 19). This variant of FREM1 was named as TILRR (Toll-like/IL-1 receptor regulator) (19). In this study, we conducted extensive analysis of genes influenced by TILRR overexpression in two cell lines, human cervical epithelial (HeLa) cells and human normal vaginal mucosal (VK2/E6E7) cells, using a PCR array system that can simultaneously interrogate the expression of 84 genes in the NFκB signal transduction pathway and RT^2^ qPCR Primer assay for selected immune and inflammation responsive genes.

The data from our study showed that TILRR overexpression significantly regulated the expression of immune and inflammation responsive genes in a dose dependent manner. Our study also showed that overexpression of TILRR up-regulated the expression of many genes in the NFκB signaling pathway, far more than previously reported. In addition to the expression of IL-1R1, MYD88, TRAF6 reported previously (19), among the 84 genes involved in the NFκB signaling pathway, TILRR overexpression significantly up-regulated the expression of many genes in HeLa and VK2/E6E7 cells. Among the significantly up-regulated genes, some have critical roles in NFκB activation, and innate and adaptive immune responses (Table S6). The effects of TILRR on these NFκB signaling related genes and inflammation mediated genes demonstrated the importance of TILRR in immune regulation and inflammatory responses. A number of studies showed that NFκB pathway coordinates the expression of several hundred functionally diverse genes in many key cellular and physiological processes, such as immune regulation, cytokine/chemokine(s) production, cell adhesion, survival and proliferation (24–28). Our data demonstrated that TILRR overexpression alone increased the expression of 1) signaling ligands such as CXCL8 (IL-8), IL-1α, IL-1β, and TNFα; 2) cell surface receptor like, TLR2, TLR3, TNFRSF10A, and TNFRSF10B, and many downstream signaling genes in NFκB signal transduction pathway (Figure 7) and significantly augmented the mRNA transcript expression of several immune responsive genes when together with IL-1β in serum free media (Figure 8). Thus, TILRR appears to not only be a co-receptor of the IL-1R1, but also have a direct effect on genes in the NFκB signal transduction and inflammation pathway.

This study also showed that as the result of TILRR overexpression the production of several inflammatory cytokine/chemokine(s) secretion was also increased. Overexpression of TILRR increased IL-6, IL-8 (CXCL8), IP-10 (CXCL10), MCP-1, and RANTES (CCL5) in the HeLa cell culture supernatants. The IL-6, IL-8 (CXCL8), IP-10 (CXCL10), MIP-1β, and RANTES (CCL5) in VK2/E6E7 cell culture supernatants were also increased. The increase in protein level of these mediators is consistent with the increase in mRNA transcript level expression. It shows that TILRR influences the production of the inflammatory mediators, although the mechanisms need to be explored in future studies. These pro-inflammatory cytokine/chemokine(s) have been reported as multifunctional, local exudation inducer, potent activator of nuclear localization of NFκB, and enhancer of inflammation (29–35). Regulation of these pro-inflammatory cytokine/chemokine(s) by TILRR suggests that TILRR may be a direct regulator in the NFκB signal transduction and inflammatory responses.

In humans, cervical epithelial cells express higher amounts of FREM1 mRNA when compared to other tissues (5). Using HeLa and VK2/E6E7 cells as an *in vitro* system in this study may help to understand the influence of TILRR, a variant of FREM1, on the inflammatory responses in the epithelial mucosal barrier. Mucosal epithelial cells not only serve as a physical barrier, but also act as the first line of defense against infection. Breaches in the epithelial lining increase the risk of inflammation and infection (36, 37).

Our study is the first to show that TILRR overexpression regulates the expression of many genes in the NFκB signal transduction pathway. TILRR could be an important mediator of NFκB signaling pathway and plays a major role in regulating innate immune and inflammatory responses and may play an important role in microbial infection and disease pathogenesis.

## Author Contributions

MK, FP, and ML: conceived and designed research; MK, HL, NT, LL, and XY: performed research; MK, HL, RO, LRL, BL, and ML: analyzed data; FP and ML: acquired funding; X-YY: supported reagents; BL, FP, and ML: supervised research; MK, HL, and ML: wrote the paper; RO, LRL, BL, and JK: edited the paper. All authors have approved this manuscript.

### Conflict of Interest Statement

The authors declare that the research was conducted in the absence of any commercial or financial relationships that could be construed as a potential conflict of interest.
