# Supplementary material for: Toll-like Interleukin 1 Receptor Regulator Is an Important Modulator of Inflammation Responsive Genes
Source: Front Immunol. 2019 Feb 28;10:272. doi: 10.3389/fimmu.2019.00272 (PMC6403165; doi:10.3389/fimmu.2019.00272)
Supplement: Supplementary file 1 [file Data_Sheet_1.pdf]

Toll-like Interleukin 1 Receptor Regulator is an important modulator of inflammation responsive genes

Mohammad Abul Kashem[1,3], Hongzhao Li[1,3], Nikki Pauline Toledo[1,3], Robert Were Omange[1,3], Binhua Liang[2,3], Lewis Ruxi Liu[1,3], Lin Li[1,3], Xuefen Yang[3], Xin-Yong Yuan[4], Jason Kindrachuk[1], Francis A Plummer[1], Ma Luo[1,3,4*]

[1]Department of Medical Microbiology and Infectious Diseases, University of Manitoba, Winnipeg, MB, Canada

[2]Department of Biochemistry & Medical Genetics, University of Manitoba, Winnipeg, MB, Canada

[3]JC Wilt Infectious Diseases Research Centre, National Microbiology Laboratory, Winnipeg, MB, Canada

[4]National Microbiology Laboratory, Public Health Agency of Canada, Winnipeg, MB, Canada.

[*]Address correspondence to: Ma Luo, PhD
JC Wilt Infectious Diseases Research Center, National Microbiology Laboratory, Winnipeg, MB, Canada; and
Department of Medical Microbiology, University of Manitoba, Winnipeg, MB, Canada
Phone: 204-789-5072, Fax: 204-789-2018
Email: ma.luo@canada.ca, Ma.Luo@umanitoba.ca

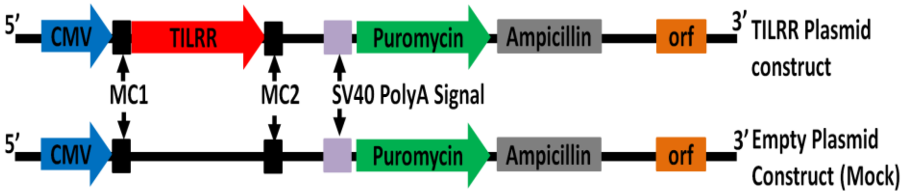

Figure S1. Diagram of plasmid constructs. TILRR (2148bp) inserted pEZ-M68 vector containing CMV promoter, puromycin and ampicillin marker, and orf backbone (top), empty control pEZ-M68 vector containing CMV promoter, puromycin and ampicillin marker, and orf (bottom).

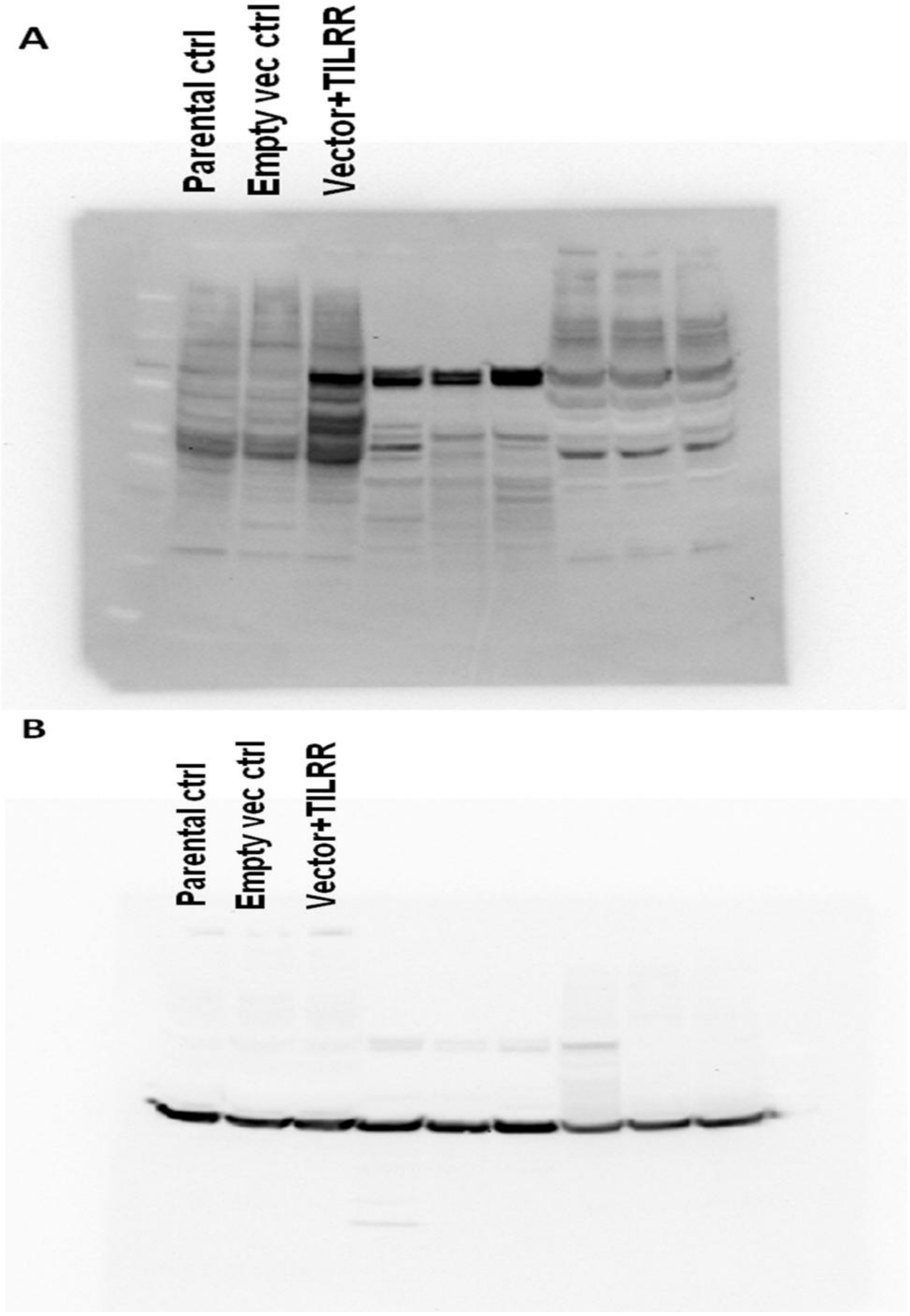

Figure S2. Full-length original blots images. (A) Western blots confirmation of TILRR overexpression in HeLa cells compared to empty vector and parental controls. (**B**) GAPDH control expression for the same HeLa samples mentioned in the figure A. Unlabelled columns are not related in this study.

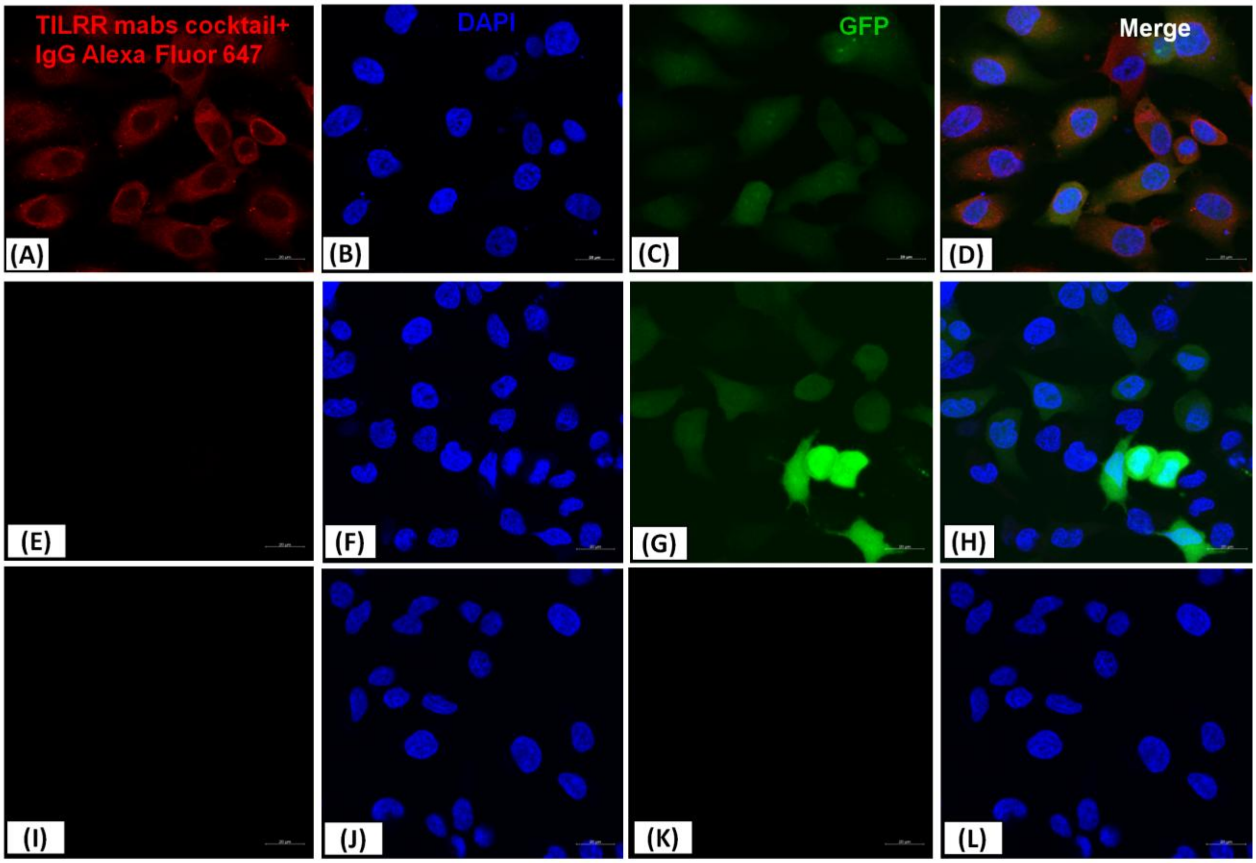

Figure S3. Overexpressed TILRR protein in transfected cells under confocal microscopy. HeLa cells were prepared and stained for confocal imaging as described in materials and method section. (**A-D)** TILRR protein expression in TILRR overexpressed cells, (**E-H)** Empty vector-transfected control cells **(I-L)** Parental non-transfected cells. Color code: red, TILRR protein (Alexa Fluor 647 channel); blue, nuclear DNA (DAPI channel); and green, eGFP (FITC channel). Image captured using 20x objectives with 20µm scale.

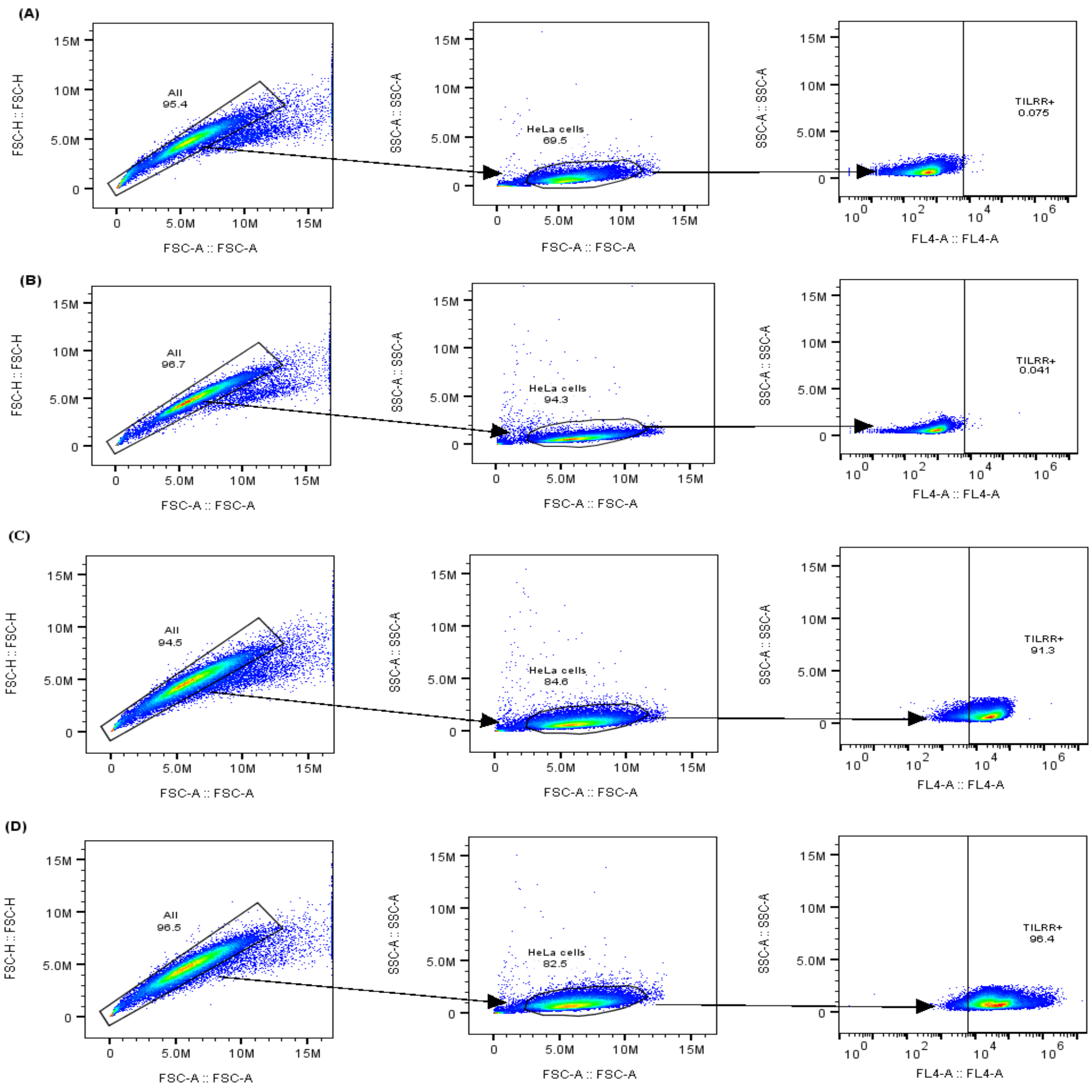

Figure S4. Gating strategy for TILRR overexpressed cells using Flow cytometry. Transfected HeLa cells were directly stained with Alexa Fluor 647 labeled in-house developed mouse anti-TILRR mabs cocktail or isotype control mab (negative) as described in materials and methods section. **(A)** Non-transfected parental base control, **(B)** TILRR-transfected cells stained with Alexa Fluor 647 labeled non-specific monoclonal antibody (isotype control), **(C)** Empty vector- and **(D)** TILRR- transfected cells stained with Alexa Fluor 647 labeled in-house developed mabs cocktail targeting epitopes of TILRR. The samples (three biological replicates for each condition) run in BD Accuri C6 flow cytometry (BD Biosciences) and data analysis done using FlowJo software (TreeStar, USA).

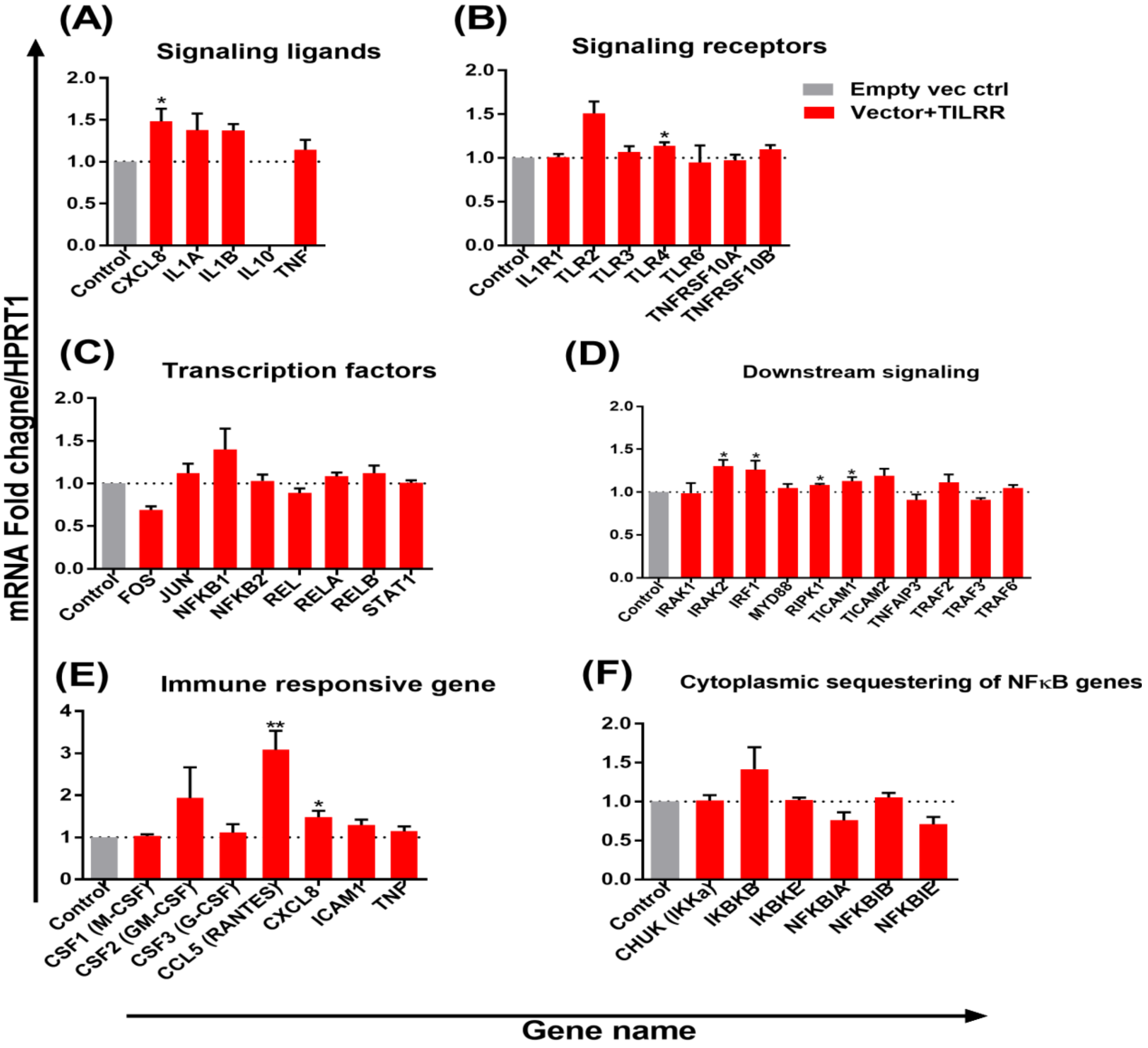

Figure S5. The mRNA transcripts fold change of NFκB signal transduction pathway after three hours incubation following transfection. HeLa cells were transiently co-transfected with either pEZ-TILRR-M68 (Vector+TILRR) (1.0µg/5x10^5 cells) or pEZ-NEG-M68 (empty vector control) (1.0µg/5x10^5 cells) along with PmaxGFP (0.2µg/5x10^5 cells) in parallel experiments for 24h at 37^0C with 5% CO_2 as described in material and methods section. The cells were incubated in serum free DMEM for 3h. Harvested RNAs from both cells used for synthesis of cDNAs that finally run with RT^2 profiler qPCR array targeting NFκB signaling components such as signaling ligands **(A),** signaling receptors **(B)**, transcription factors **(C),** downstream signaling genes **(D)**, immune responsive genes **(E)**, and cytoplasmic sequestering of NFκB genes **(F).** Data were analyzed using GeneGlobe Data Analysis Centre (Qiagen) for RT^2 profiler PCR array. Fold induction for individual gene in TILRR transfected cells expressed as relative to levels of empty vector transfected control and show mean±SEM of three independent experiments. All data were normalized against HPRT1 housekeeping gene. The statistical comparisons conducted using student t test, all $p<0.05$ were reported and indicated using an asterisks' *$p<0.05$, and **$p<0.01$. Legends on the upper right corner represent the experimental conditions.

Table S1. Dose response effect of TILRR on immune responsive genes in HeLa cells

Gene name	0.25 µg DNA		0.5 µg DNA		1.0 µg DNA		2.0 µg DNA	
	Fold Increase (FI) (mean±SEM)	p-value	FI (mean±SEM)	p-value	FI (mean±SEM)	p-value	FI (mean±SEM)	p-value
CCL5(RANTES)	3.66±0.72	0.0013	3.66±0.58	0.0006	7.76±0.90	0.0031	6.26±1.20	0.0009
CXCL8 (IL-8)	2.16±0.61	0.0122	10.39±2.00	0.0012	11.28±2.69	0.0121	6.98±1.71	0.0001
IL-6	1.52±0.16	0.0091	5.35±0.14	0.0017	5.52±1.30	0.0005	4.75±0.64	0.0003
TNFα	2.08±1.12	0.1651	3.21±0.24	0.0010	5.63±3.69	0.0100	3.63±2.50	0.0171

Table S2. Dose response effect of TILRR on immune responsive genes in VK2/E6E7 cells

Gene name	0.25 µg DNA		0.5 µg DNA		1.0 µg DNA		2.0 µg DNA	
	FI (mean±SEM)	p-value	FI (mean±SEM)	p-value	FI (mean±SEM)	p-value	FI (mean±SEM)	p-value
CCL5(RANTES)	1.05±0.05	0.1857	1.18±0.22	0.1536	1.07±0.30	0.6925	1.01±0.17	0.9736
CXCL8 (IL-8)	0.93±0.13	0.6583	1.86±0.05	0.0020	1.88±0.90	0.0286	1.27±0.10	0.0066
IL-6	1.03±0.11	0.4043	1.59±0.31	0.0238	2.47±0.40	0.0031	0.98±0.23	0.8544
TNFα	0.89±0.16	0.2945	1.42±0.37	0.0775	2.40±1.39	0.0919	1.32±0.55	0.2911

Table S3. Gene categories (PAHS-025Z, Qiagen)

Category	Gene
NFκB Signaling Ligands	CXCL8(IL-8), IL-1A, IL-1B, IL-10, TNF
NFκB Signaling receptors	CD27 (TNFRSF7), CD40 (TNFRSF5), EGFR (ERBB1), F2R (PAR1), FASLG (TNFSF6), IL-1R1, LTBR, NOD1 (CARD4), TLR1, TLR2, TLR3, TLR4, TLR6, TLR9, TNFRSF1A (TNFR1), TNFRSF10A (TRAIL-R), TNFRSF10B (DR5), TNFSF10 (TRAIL), TNFSF14
NFκB Transcription Factors	ATF1, EGR1, ELK1, FOS, JUN, STAT1, NFKB1 (p105/p50), NFKB2 (p100/p52), REL (p65), RELA, RELB
Signaling Downstream of NFκB	BIRC2 (cIAP1), FADD, IRAK1, IRAK2, IRF1, MYD88, RIPK1, TBK1, TICAM1 (TRIF), TICAM2 (TRAM), TNFAIP3, TRADD, TRAF2, TRAF3, TRAF6
Immune Responsive genes	CCL2 (MCP-1), CCL5 (RANTES), CSF1 (M-CSF), CSF2 (GM-CSF), CSF3 (G-CSF), CXCL8 (IL-8), ICAM1, IFNA1, IFNG, LTA (TNFB), TNF
Cytoplasmic Sequestering / Releasing of NFκB	BCL3, CHUK (IKKα), IKBKB (IKKβ), IKBKE (IKKε), IKBKG, NFKBIA (IκBα), NFKBIB (IκBβ), NFKBIE
Apoptosis	AGT, BCL2A1 (BFL1), BCL2L1 (BCLXL), BIRC3 (c-IAP2)
Other NFκB Signaling	AKT1, MAP3K1 (MEKK1), RAF1, BCL10, CARD11, CASP1 (ICE), CASP8 (FLICE), CFLAR (Casper), HMOX1, MALT1, PSIP1, RHOA, TIMP1

Table S4. Detailed description of genes associated with NFκB signaling pathway (PAHS-025Z, Qiagen)

Gene Symbol	Descriptions	UniGene	GenBank
AGT	Angiotensinogen (serpin peptidase inhibitor, clade A, member 8)	Hs.19383	NM_000029
AKT1	V-akt murine thymoma viral oncogene homolog 1	Hs.525622	NM_005163
ATF1	Activating transcription factor 1	Hs.648565	NM_005171
BCL10	B-cell CLL/lymphoma 10	Hs.193516	NM_003921
BCL2A1	BCL2-related protein A1	Hs.227817	NM_004049
BCL2L1	BCL2-like 1	Hs.516966	NM_138578
BCL3	B-cell CLL/lymphoma 3	Hs.31210	NM_005178
BIRC2	Baculoviral IAP repeat containing 2	Hs.696238	NM_001166
BIRC3	Baculoviral IAP repeat containing 3	Hs.127799	NM_001165
CARD11	Caspase recruitment domain family, member 11	Hs.648101	NM_032415
CASP1	Caspase 1, apoptosis-related cysteine peptidase (interleukin 1, beta, convertase)	Hs.2490	NM_033292
CASP8	Caspase 8, apoptosis-related cysteine peptidase	Hs.599762	NM_001228
CCL2	Chemokine (C-C motif) ligand 2	Hs.303649	NM_002982
CCL5	Chemokine (C-C motif) ligand 5	Hs.514821	NM_002985
CD27	CD27 molecule	Hs.355307	NM_001242
CD40	CD40 molecule, TNF receptor superfamily member 5	Hs.472860	NM_001250
CFLAR	CASP8 and FADD-like apoptosis regulator	Hs.390736	NM_003879
CHUK	Conserved helix-loop-helix ubiquitous kinase	Hs.198998	NM_001278
CSF1	Colony stimulating factor 1 (macrophage)	Hs.591402	NM_000757
CSF2	Colony stimulating factor 2 (granulocyte-macrophage)	Hs.1349	NM_000758
CSF3	Colony stimulating factor 3 (granulocyte)	Hs.2233	NM_000759
EGFR	Epidermal growth factor receptor	Hs.488293	NM_005228
EGR1	Early growth response 1	Hs.326035	NM_001964
ELK1	ELK1, member of ETS oncogene family	Hs.181128	NM_005229
F2R	Coagulation factor II (thrombin) receptor	Hs.482562	NM_001992
FADD	Fas (TNFRSF6)-associated via death domain	Hs.86131	NM_003824
FASLG	Fas ligand (TNF superfamily, member 6)	Hs.2007	NM_000639
FOS	FBJ murine osteosarcoma viral oncogene homolog	Hs.728789	NM_005252
HMOX1	Heme oxygenase (decycling) 1	Hs.517581	NM_002133

ICAM1	Intercellular adhesion molecule 1	Hs.643447	NM_000201
IFNA1	Interferon, alpha 1	Hs.37026	NM_024013
IFNG	Interferon, gamma	Hs.856	NM_000619
IKBKB	Inhibitor of kappa light polypeptide gene enhancer in B-cells, kinase beta	Hs.597664	NM_001556
IKBKE	Inhibitor of kappa light polypeptide gene enhancer in B-cells, kinase epsilon	Hs.321045	NM_014002
IKBKG	Inhibitor of kappa light polypeptide gene enhancer in B-cells, kinase gamma	Hs.43505	NM_003639
IL10	Interleukin 10	Hs.193717	NM_000572
IL1A	Interleukin 1, alpha	Hs.1722	NM_000575
IL1B	Interleukin 1, beta	Hs.126256	NM_000576
IL1R1	Interleukin 1 receptor, type I	Hs.701982	NM_000877
CXCL8	Interleukin 8	Hs.624	NM_000584
IRAK1	Interleukin-1 receptor-associated kinase 1	Hs.522819	NM_001569
IRAK2	Interleukin-1 receptor-associated kinase 2	Hs.449207	NM_001570
IRF1	Interferon regulatory factor 1	Hs.436061	NM_002198
JUN	Jun proto-oncogene	Hs.714791	NM_002228
LTA	Lymphotoxin alpha (TNF superfamily, member 1)	Hs.36	NM_000595
LTBR	Lymphotoxin beta receptor (TNFR superfamily, member 3)	Hs.1116	NM_002342
MALT1	Mucosa associated lymphoid tissue lymphoma translocation gene 1	Hs.601217	NM_173844
MAP3K1	Mitogen-activated protein kinase kinase kinase 1	Hs.657756	NM_005921
MYD88	Myeloid differentiation primary response gene (88)	Hs.82116	NM_002468
NFKB1	Nuclear factor of kappa light polypeptide gene enhancer in B-cells 1	Hs.654408	NM_003998
NFKB2	Nuclear factor of kappa light polypeptide gene enhancer in B-cells 2 (p49/p100)	Hs.73090	NM_002502
NFKBIA	Nuclear factor of kappa light polypeptide gene enhancer in B-cells inhibitor, alpha	Hs.81328	NM_020529
NFKBIB	Nuclear factor of kappa light polypeptide gene enhancer in B-cells inhibitor, beta	Hs.9731	NM_002503
NFKBIE	Nuclear factor of kappa light polypeptide gene enhancer in B-cells inhibitor, epsilon	Hs.458276	NM_004556
NOD1	Nucleotide-binding oligomerization domain containing 1	Hs.405153	NM_006092
PSIP1	PC4 and SFRS1 interacting protein 1	Hs.658434	NM_021144
RAF1	V-raf-1 murine leukemia viral oncogene homolog 1	Hs.159130	NM_002880
REL	V-rel reticuloendotheliosis viral oncogene homolog (avian)	Hs.631886	NM_002908
RELA	V-rel reticuloendotheliosis viral oncogene homolog A (avian)	Hs.502875	NM_021975
RELB	V-rel reticuloendotheliosis viral oncogene homolog B	Hs.654402	NM_006509
RHOA	Ras homolog gene family, member A	Hs.247077	NM_001664
RIPK1	Receptor (TNFRSF)-interacting serine-threonine kinase 1	Hs.519842	NM_003804

STAT1	Signal transducer and activator of transcription 1, 91kDa	Hs.642990	NM_007315
TBK1	TANK-binding kinase 1	Hs.505874	NM_013254
TICAM1	Toll-like receptor adaptor molecule 1	Hs.29344	NM_182919
TICAM2	Toll-like receptor adaptor molecule 2	Hs.710895	NM_021649
TIMP1	TIMP metallopeptidase inhibitor 1	Hs.522632	NM_003254
TLR1	Toll-like receptor 1	Hs.654532	NM_003263
TLR2	Toll-like receptor 2	Hs.519033	NM_003264
TLR3	Toll-like receptor 3	Hs.657724	NM_003265
TLR4	Toll-like receptor 4	Hs.174312	NM_138554
TLR6	Toll-like receptor 6	Hs.662185	NM_006068
TLR9	Toll-like receptor 9	Hs.87968	NM_017442
TNF	Tumor necrosis factor	Hs.241570	NM_000594
TNFAIP3	Tumor necrosis factor, alpha-induced protein 3	Hs.211600	NM_006290
TNFRSF10A	Tumor necrosis factor receptor super family, member 10a	Hs.591834	NM_003844
TNFRSF10B	Tumor necrosis factor receptor super family, member 10b	Hs.521456	NM_003842
TNFRSF1A	Tumor necrosis factor receptor superfamily, member 1A	Hs.279594	NM_001065
TNFSF10	Tumor necrosis factor (ligand) superfamily, member 10	Hs.478275	NM_003810
TNFSF14	Tumor necrosis factor (ligand) superfamily, member 14	Hs.129708	NM_003807
TRADD	TNFRSF1A-associated via death domain	Hs.460996	NM_003789
TRAF2	TNF receptor-associated factor 2	Hs.522506	NM_021138
TRAF3	TNF receptor-associated factor 3	Hs.510528	NM_003300
TRAF6	TNF receptor-associated factor 6	Hs.591983	NM_004620
ACTB	Actin, beta	Hs.520640	NM_001101
B2M	Beta-2-microglobulin	Hs.534255	NM_004048
GAPDH	Glyceraldehyde-3-phosphate dehydrogenase	Hs.592355	NM_002046
HPRT1	Hypoxanthine phosphoribosyltransferase 1	Hs.412707	NM_000194
RPLP0	Ribosomal protein, large, P0	Hs.546285	NM_001002

Table S5: List of primary and secondary antibodies used in Bioplex Multiplex cytokine/chemokine(s) bead assay

**Primary antibody**			
**SL**	**Name**	**Catalog#**	**Vendor**
1	Human CXCL8/IL-8 MAb	M801	ThermoFisher Scientific
2	Human CCL5/RANTES PAb	P230E	ThermoFisher Scientific
3	Rat Anti-Human GM-CSF-UNLB	10111-01	SouthernBiotech
4	Human IFNγ MAb	M700A	ThermoFisher Scientific
5	Human IL-1beta/ IL-1F2 Antibody	MAB601-500	R&D System
6	Human IL-6 MAb	M620	ThermoFisher Scientific
7	Rat Anti-Human IL-10-UNLB	10100-01	SouthernBiotech
8	Human/Primate IL-17/IL-17A Antibody	MAB317-500	R&D System
9	Human IP-10/ CXCL10/CRG-2 Antibody	MAB266-500	R&D System
10	Human MCP-1/CCL2/JE Antibody	MAB679-500	R&D System
11	Human MIP-1α/CCL3Antibody	AF-270-NA	R&D System
12	Human MIP-1β/CCL4 Antibody	MAB271-100	R&D System
13	Human TNFα MAb	M303	ThermoFisher Scientific
**Secondary antibody (Biotinylated)**			
**SL**	**Name**	**Catalog#**	**Vendor**
1	Human CXCL8/IL-8 MAb, Biotin-labeled	M802B	ThermoFisher Scientific
2	Human CCL5/RANTES MAb, Biotin-labeled	M230B	ThermoFisher Scientific
3	Rat Anti-Human GM-CSF-BIOT	10112-08	SouthernBiotech
4	Human IFNγ MAb, Biotin-labeled	M701B	ThermoFisher Scientific
5	Human IL-1beta/IL-1F2 Biotinylated Antibody	BAF201	R&D System
6	Human IL-6 MAb, Biotin-labeled	M621B	ThermoFisher Scientific
7	Rat Anti-Human IL-10-BIOT	10110-08	SouthernBiotech
8	Human/Primate IL-17/IL-17A Biotinylated Antibody	BAF317	R&D System
9	Human IP-10/CXCL10/CRG-2 Biotinylated Antibody	BAF266	R&D System
10	Human MCP-1/CCL2/JE Biotinylated Antibody	BAF279	R&D System
11	Human MIP-1α/CCL3 Biotinylated Antibody	BAF270	R&D System
12	Human MIP-1β/CCL4 Biotinylated Antibody	BAF271	R&D System
13	Human TNFα MAb, Biotin-labeled	M302B	ThermoFisher Scientific

Table S6. Functions of gene associated with innate immunity and inflammatory responses

Gene name	Function(s)
BCL10	Positive regulator to activate NFκB (Ruland et al., 2001, Sun and Yang, 2010)
CARD11	When expressed in cells, this protein activated NFκB (Bertin et al., 2001)
CCL2 (MCP-1)	Involved in immunoregulatory and inflammatory processes (Donadelli et al., 2000, Mezzano et al., 2004)
CCL5 (RANTES)	Activator of NFkB (Huang et al., 2009)
CHUK/IKKα	Activator of transcription factor (Adli et al., 2010, Israel, 2010, Hacker and Karin, 2006)
CSF1 (M-CSF)	Controls the production, differentiation and function of monocytes and macrophages (Stanley et al., 1997)
CSF2 (GM-CSF)	Activates monocytes/macrophages, and acts as an important hematopoietic growth factor and immune modulator (Ushach and Zlotnik, 2016, Shi et al., 2006)
CSF3 (G-CSF)	Controls the production, differentiation and function of granulocytes (Mehta et al., 2015, Xu et al., 2000)
CXCL8 (IL-8)	Major mediators associated with inflammation, plays key role in neutrophil recruitment and neutrophil degranulation (Harada et al., 1994)
IL1A	Pleiotropic cytokine involved in various immune responses and inflammatory processes (Di Paolo and Shayakhmetov, 2016, Borthwick, 2016)
IL1B	Important mediator of the inflammatory response (Di Paolo and Shayakhmetov, 2016, Borthwick, 2016)
IL1R1	Important mediator involved in many cytokine-induced immune and inflammatory responses (Gasse et al., 2007, Parpaleix et al., 2016)
IKBKB (IKKβ)	Phosphorylates the inhibitor in the inhibitor/NFκB complex, causing dissociation of the inhibitor and activation of NFκB (Adli et al., 2010, Israel, 2010, Hacker and Karin, 2006, De Molfetta et al., 2010)
IRAK1	Partially responsible for IL1-induced upregulation of the transcription factor NFκB (Liu et al., 2008)
IRAK2	Participates in the IL1-induced upregulation of the transcription factor NFκB (Keating et al., 2007, Muzio et al., 1997)
MALT1	Activate NFκB (Kingeter and Schaefer, 2010, Sun and Yang, 2010)
MYD88	Plays a central role in the innate and adaptive immune response, functions as essential signal transducer in the IL-1 and TLR signaling pathways (Gasse et al., 2007, Parpaleix et al., 2016, Muzio et al., 1997)
NFKB1 (p105/p50)	Activated NFκB1 translocates in to the nucleus and stimulates the expression of genes involved in a wide variety of biological functions (Yu et al., 2009, Lawrence, 2009)
NFKB2 (p100/p52)	Involves in non-canonical activation of NFκB pathway (Sun, 2012, Sun, 2011, Lawrence, 2009)

NFKBIA/IkBα	Inhibits NFκB/REL complexes, which are involved in inflammatory responses (Verma et al., 1995, Jacobs and Harrison, 1998)
NFKBIB/IkBβ	Inhibits NFκB by complexing with and trapping it in the cytoplasm (Woronicz et al., 1997)
RAF1	Activates NFκB transcription factor (Yeung et al., 2001, Li and Sedivy, 1993)
REL	Involves in NFκB activation, immune response and inflammation (Oeckinghaus and Ghosh, 2009)
TBK1	Mediates NFκB activation (Pomerantz and Baltimore, 1999, Moser et al., 2015)
TICAM1 (TRIF)	Involves in native immunity against invading pathogens (Funami et al., 2008)
TLR1, TLR2, TLR3, TLR4 and TLR6	Plays a fundamental role in pathogen recognition and activation of innate immunity, Activation of TLRs leads to the up-regulation of signaling pathways to modulate the host's inflammatory response (Alexopoulou et al., 2001, Yu and Levine, 2011, Barton and Kagan, 2009, O'Neill et al., 2013, O'Neill, 2006)
TNF	Regulate wide spectrum of biological process including cell proliferation, differentiation and apoptosis (Schutze et al., 1995, Zhou et al., 2003, Gupta et al., 2005)
TRAF2	Mediates the signal transduction from members of the TNF receptor superfamily. Required for TNF-alpha-mediated activation of NFkB (Pomerantz and Baltimore, 1999, Zhang et al., 2011, Etemadi et al., 2015, Baud et al., 1999)
TRAF3	Participates in the signal transduction of CD40 and important for the activation of the immune response (Ni et al., 2000)
TRAF6	Functions as a signal transducer in the NFkB pathway (Sun and Yang, 2010, Baud et al., 1999, Walsh et al., 2008)

Supplementary References

ADLI, M., MERKHOFER, E., COGSWELL, P. & BALDWIN, A. S. (2010). IKKalpha and IKKbeta each function to regulate NF-kappaB activation in the TNF-induced/canonical pathway. *PLoS One* 5(2), e9428.

ALEXOPOULOU, L., HOLT, A. C., MEDZHITOV, R. & FLAVELL, R. A. (2001). Recognition of double-stranded RNA and activation of NF-kappaB by Toll-like receptor 3. *Nature* 413(6857), 732-8.

BARTON, G. M. & KAGAN, J. C. (2009). A cell biological view of Toll-like receptor function: regulation through compartmentalization. *Nat Rev Immunol* 9(8), 535-42.

BAUD, V., LIU, Z. G., BENNETT, B., SUZUKI, N., XIA, Y. & KARIN, M. (1999). Signaling by proinflammatory cytokines: oligomerization of TRAF2 and TRAF6 is sufficient for JNK and IKK activation and target gene induction via an amino-terminal effector domain. *Genes Dev* 13(10), 1297-308.

BERTIN, J., WANG, L., GUO, Y., JACOBSON, M. D., POYET, J. L., SRINIVASULA, S. M., et al. (2001). CARD11 and CARD14 are novel caspase recruitment domain (CARD)/membrane-associated guanylate kinase (MAGUK) family members that interact with BCL10 and activate NF-kappa B. *J Biol Chem* 276(15), 11877-82.

BORTHWICK, L. A. (2016). The IL-1 cytokine family and its role in inflammation and fibrosis in the lung. *Semin Immunopathol* 38(4), 517-34.

DE MOLFETTA, G. A., LUCIOLA ZANETTE, D., ALEXANDRE PANEPUCCI, R., DOS SANTOS, A. R., DA SILVA, W. A., JR. & ANTONIO ZAGO, M. (2010). Role of NFKB2 on the early myeloid differentiation of CD34+ hematopoietic stem/progenitor cells. *Differentiation* 80(4-5), 195-203.

DI PAOLO, N. C. & SHAYAKHMETOV, D. M. (2016). Interleukin 1alpha and the inflammatory process. *Nat Immunol* 17(8), 906-13.

DONADELLI, R., ABBATE, M., ZANCHI, C., CORNA, D., TOMASONI, S., BENIGNI, A., et al. (2000). Protein traffic activates NF-kB gene signaling and promotes MCP-1-dependent interstitial inflammation. *Am J Kidney Dis* 36(6), 1226-41.

ETEMADI, N., CHOPIN, M., ANDERTON, H., TANZER, M. C., RICKARD, J. A., ABEYSEKERA, W., et al. (2015). TRAF2 regulates TNF and NF-kappaB signalling to suppress apoptosis and skin inflammation independently of Sphingosine kinase 1. *Elife* 4.

FUNAMI, K., SASAI, M., OSHIUMI, H., SEYA, T. & MATSUMOTO, M. (2008). Homo-oligomerization is essential for Toll/interleukin-1 receptor domain-containing adaptor molecule-1-mediated NF-kappaB and interferon regulatory factor-3 activation. *J Biol Chem* 283(26), 18283-91.

GASSE, P., MARY, C., GUENON, I., NOULIN, N., CHARRON, S., SCHNYDER-CANDRIAN, S., et al. (2007). IL-1R1/MyD88 signaling and the inflammasome are essential in pulmonary inflammation and fibrosis in mice. *J Clin Invest* 117(12), 3786-99.

GUPTA, S., BI, R., KIM, C., CHIPLUNKAR, S., YEL, L. & GOLLAPUDI, S. (2005). Role of NF-kappaB signaling pathway in increased tumor necrosis factor-alpha-induced apoptosis of lymphocytes in aged humans. *Cell Death Differ* 12(2), 177-83.

HACKER, H. & KARIN, M. (2006). Regulation and function of IKK and IKK-related kinases. *Sci STKE* 2006(357), re13.

HARADA, A., SEKIDO, N., AKAHOSHI, T., WADA, T., MUKAIDA, N. & MATSUSHIMA, K. (1994). Essential involvement of interleukin-8 (IL-8) in acute inflammation. *J Leukoc Biol* 56(5), 559-64.

HUANG, C. Y., FONG, Y. C., LEE, C. Y., CHEN, M. Y., TSAI, H. C., HSU, H. C., et al. (2009). CCL5 increases lung cancer migration via PI3K, Akt and NF-kappaB pathways. *Biochem Pharmacol* 77(5), 794-803.

ISRAEL, A. (2010). The IKK complex, a central regulator of NF-kappaB activation. *Cold Spring Harb Perspect Biol* 2(3), a000158.

JACOBS, M. D. & HARRISON, S. C. (1998). Structure of an IkappaBalpha/NF-kappaB complex. *Cell* 95(6), 749-58.

KEATING, S. E., MALONEY, G. M., MORAN, E. M. & BOWIE, A. G. (2007). IRAK-2 participates in multiple toll-like receptor signaling pathways to NFkappaB via activation of TRAF6 ubiquitination. *J Biol Chem* 282(46)**,** 33435-43.

KINGETER, L. M. & SCHAEFER, B. C. (2010). Malt1 and cIAP2-Malt1 as effectors of NF-kappaB activation: kissing cousins or distant relatives? *Cell Signal* 22(1)**,** 9-22.

LAWRENCE, T. (2009). The nuclear factor NF-kappaB pathway in inflammation. *Cold Spring Harb Perspect Biol* 1(6)**,** a001651.

LI, S. & SEDIVY, J. M. (1993). Raf-1 protein kinase activates the NF-kappa B transcription factor by dissociating the cytoplasmic NF-kappa B-I kappa B complex. *Proc Natl Acad Sci U S A* 90(20)**,** 9247-51.

LIU, G., PARK, Y. J. & ABRAHAM, E. (2008). Interleukin-1 receptor-associated kinase (IRAK) -1-mediated NF-kappaB activation requires cytosolic and nuclear activity. *FASEB J* 22(7)**,** 2285-96.

MEHTA, H. M., MALANDRA, M. & COREY, S. J. (2015). G-CSF and GM-CSF in Neutropenia. *J Immunol* 195(4)**,** 1341-9.

MEZZANO, S., AROS, C., DROGUETT, A., BURGOS, M. E., ARDILES, L., FLORES, C., et al. (2004). NF-kappaB activation and overexpression of regulated genes in human diabetic nephropathy. *Nephrol Dial Transplant* 19(10)**,** 2505-12.

MOSER, C. V., STEPHAN, H., ALTENRATH, K., KYNAST, K. L., RUSSE, O. Q., OLBRICH, K., et al. (2015). TANK-binding kinase 1 (TBK1) modulates inflammatory hyperalgesia by regulating MAP kinases and NF-kappaB dependent genes. *J Neuroinflammation* 12**,** 100.

MUZIO, M., NI, J., FENG, P. & DIXIT, V. M. (1997). IRAK (Pelle) family member IRAK-2 and MyD88 as proximal mediators of IL-1 signaling. *Science* 278(5343)**,** 1612-5.

NI, C. Z., WELSH, K., LEO, E., CHIOU, C. K., WU, H., REED, J. C., et al. (2000). Molecular basis for CD40 signaling mediated by TRAF3. *Proc Natl Acad Sci U S A* 97(19)**,** 10395-9.

O'NEILL, L. A. (2006). How Toll-like receptors signal: what we know and what we don't know. *Curr Opin Immunol* 18(1)**,** 3-9.

O'NEILL, L. A., GOLENBOCK, D. & BOWIE, A. G. (2013). The history of Toll-like receptors - redefining innate immunity. *Nat Rev Immunol* 13(6)**,** 453-60.

OECKINGHAUS, A. & GHOSH, S. (2009). The NF-kappaB family of transcription factors and its regulation. *Cold Spring Harb Perspect Biol* 1(4)**,** a000034.

PARPALEIX, A., AMSELLEM, V., HOUSSAINI, A., ABID, S., BREAU, M., MARCOS, E., et al. (2016). Role of interleukin-1 receptor 1/MyD88 signalling in the development and progression of pulmonary hypertension. *Eur Respir J* 48(2)**,** 470-83.

POMERANTZ, J. L. & BALTIMORE, D. (1999). NF-kappaB activation by a signaling complex containing TRAF2, TANK and TBK1, a novel IKK-related kinase. *EMBO J* 18(23)**,** 6694-704.

RULAND, J., DUNCAN, G. S., ELIA, A., DEL BARCO BARRANTES, I., NGUYEN, L., PLYTE, S., et al. (2001). Bcl10 is a positive regulator of antigen receptor-induced activation of NF-kappaB and neural tube closure. *Cell* 104(1)**,** 33-42.

SCHUTZE, S., WIEGMANN, K., MACHLEIDT, T. & KRONKE, M. (1995). TNF-induced activation of NF-kappa B. *Immunobiology* 193(2-4)**,** 193-203.

SHI, Y., LIU, C. H., ROBERTS, A. I., DAS, J., XU, G., REN, G., et al. (2006). Granulocyte-macrophage colony-stimulating factor (GM-CSF) and T-cell responses: what we do and don't know. *Cell Res* 16(2)**,** 126-33.

STANLEY, E. R., BERG, K. L., EINSTEIN, D. B., LEE, P. S., PIXLEY, F. J., WANG, Y., et al. (1997). Biology and action of colony--stimulating factor-1. *Mol Reprod Dev* 46(1)**,** 4-10.

SUN, S. C. (2011). Non-canonical NF-kappaB signaling pathway. *Cell Res* 21(1)**,** 71-85.

SUN, S. C. (2012). The noncanonical NF-kappaB pathway. *Immunol Rev* 246(1)**,** 125-40.

SUN, W. & YANG, J. (2010). Molecular basis of lysophosphatidic acid-induced NF-kappaB activation. *Cell Signal* 22(12)**,** 1799-803.

USHACH, I. & ZLOTNIK, A. (2016). Biological role of granulocyte macrophage colony-stimulating factor (GM-CSF) and macrophage colony-stimulating factor (M-CSF) on cells of the myeloid lineage. *J Leukoc Biol* 100(3)**,** 481-9.

VERMA, I. M., STEVENSON, J. K., SCHWARZ, E. M., VAN ANTWERP, D. & MIYAMOTO, S. (1995). Rel/NF-kappa B/I kappa B family: intimate tales of association and dissociation. *Genes Dev* 9(22)**,** 2723-35.

WALSH, M. C., KIM, G. K., MAURIZIO, P. L., MOLNAR, E. E. & CHOI, Y. (2008). TRAF6 autoubiquitination-independent activation of the NFkappaB and MAPK pathways in response to IL-1 and RANKL. *PLoS One* 3(12)**,** e4064.

WORONICZ, J. D., GAO, X., CAO, Z., ROTHE, M. & GOEDDEL, D. V. (1997). IkappaB kinase-beta: NF-kappaB activation and complex formation with IkappaB kinase-alpha and NIK. *Science* 278(5339)**,** 866-9.

XU, S., HOGLUND, M., HAKANSSON, L. & VENGE, P. (2000). Granulocyte colony-stimulating factor (G-CSF) induces the production of cytokines in vivo. *Br J Haematol* 108(4)**,** 848-53.

YEUNG, K. C., ROSE, D. W., DHILLON, A. S., YAROS, D., GUSTAFSSON, M., CHATTERJEE, D., et al. (2001). Raf kinase inhibitor protein interacts with NF-kappaB-inducing kinase and TAK1 and inhibits NF-kappaB activation. *Mol Cell Biol* 21(21)**,** 7207-17.

YU, M. & LEVINE, S. J. (2011). Toll-like receptor, RIG-I-like receptors and the NLRP3 inflammasome: key modulators of innate immune responses to double-stranded RNA viruses. *Cytokine Growth Factor Rev* 22(2)**,** 63-72.

YU, Y., WAN, Y. & HUANG, C. (2009). The biological functions of NF-kappaB1 (p50) and its potential as an anti-cancer target. *Curr Cancer Drug Targets* 9(4)**,** 566-71.

ZHANG, L., BLACKWELL, K., ALTAEVA, A., SHI, Z. & HABELHAH, H. (2011). TRAF2 phosphorylation promotes NF-kappaB-dependent gene expression and inhibits oxidative stress-induced cell death. *Mol Biol Cell* 22(1)**,** 128-40.

ZHOU, A., SCOGGIN, S., GAYNOR, R. B. & WILLIAMS, N. S. (2003). Identification of NF-kappa B-regulated genes induced by TNFalpha utilizing expression profiling and RNA interference. *Oncogene* 22(13)**,** 2054-64.
